# Effectiveness of unguided web-based interventions for the treatment of depressive symptoms in improving the quality of life: a meta-analysis

**DOI:** 10.1007/s11136-025-04057-z

**Published:** 2025-10-10

**Authors:** Felix Butt, Jan-Philipp Klein, Cora Schefft, Stephan Köhler

**Affiliations:** 1 Charité – Universitätsmedizin, Berlin, Germany; 2 Universität zu Lübeck, Lübeck, Germany

**Keywords:** Quality of life, Internet interventions, Unguided, Depression, Meta-analysis

## Abstract

**Purpose:**

Unguided internet-based interventions (IBIs) have proven to be effective in reducing depressive symptoms. The primary objective of this meta-analysis was to evaluate the effect of unguided IBIs on quality of life (QoL).

**Method:**

We searched eligible databases via PubMed and OVID for articles published between the inception of the databases and the 1st of October 2024. For quality assessment, the RoB-2 tool was used. We included randomized controlled trials (RCTs) that examined unguided IBIs, specifically designed to reduce depressive symptoms for patients between the age of 18–65 years. A three-level random effects model was employed for analysing global QoL as well as mental and physical health related QoL and depressive symptoms.

**Results:**

In total, 15 studies on 20 unique IBIs were included, comprising a total sample size of 3623 participants. Unguided IBIs significantly improved users’ global QoL at the end of the intervention compared to control groups (g = 0.29, 95% CI [0.04, 0.55], *p *= 0.03). Mental and physical health QoL measurements did not significantly differ post intervention or 6-months follow-up. Furthermore, unguided IBIs had a small effect on depressive symptoms post intervention (g = − 0.37 (95% CI −0.62 to − 0.12, *p* = 0.01) but not at 6-months follow-up.

**Conclusion:**

The findings indicate that unguided IBIs are effective in enhancing users’ global QoL by the end of the intervention and in reducing depressive symptoms. The main limitations of this study are the small sample size and concerns regarding most bias domains. Further research is needed to investigate the effect of unguided IBIs on different QoL domains. Nonetheless, this meta-analysis provides valuable insights into the potential of unguided IBIs to enhance quality of life.

## Abstract (plain language summary)

While unguided internet-based interventions (IBIs) have been shown to help reduce depressive symptoms, their impact on quality of life (QoL) is still unclear. Since improving QoL is a key goal in mental health treatment, it is important to understand whether these interventions provide benefits beyond symptom reduction. This study investigates whether unguided IBIs not only help reduce depressive symptoms but also improve users' overall quality of life. The analysis included 15 studies with a total of 3623 participants The meta-analysis shows that unguided IBIs can lead to small improvements in overall QoL and help reduce depressive symptoms. However, their impact on specific aspects of QoL, such as mental and physical health, is less clear. These results suggest that while unguided IBIs can be beneficial, their impact is limited, and more research is needed to understand how they influence different areas of QoL.

## Introduction

Depression stands as one of the most prevalent illnesses worldwide, with a 12-month prevalence of approximately 5% among adults [1]. Over the past two decades, its prevalence has steadily increased [2]. Research highlights its detrimental effects on quality of life (QoL) [3]. Around 63% of patients with a major depressive episode, 85% of those with chronic or double depression, and 56% with dysthymia report QoL scores at least two standard deviations below their community average [4]. QoL is a broad construct encompassing physical, psychological, social, and environmental factors [5]. Many depressive symptoms are closely tied to QoL. Fatigue, sleep disturbances, and exhaustion impact physical QoL [6], while psychological aspects, such as reduced positive emotions, cognitive decline [7], low self-esteem [8], and body dissatisfaction [9], further impair well-being. Social and environmental factors also play a role: people with depression report poorer living conditions [10] and perceive their life as less advantageous, even when objective conditions suggest otherwise [11]. QoL is a predictor of relapse and recovery for patients with depression [12], highlighting the reciprocal relationship between depression and QoL [13]. This emphasizes the need to look beyond symptom reduction in depression treatment. Measuring symptom severity alone does not fully capture well-being; it is only one aspect among many [14]. Broader domains such as physical health, social functioning, and environmental factors remain overlooked when focusing solely on symptom reduction. Reducing symptoms does not necessarily translate to improved daily functioning or life satisfaction [13, 14]. The QoL domains are distinct in their relationship to depression. Recent studies show that depression predicts mental health QoL more strongly than other domains, though all domains are affected. Gonzales-Blanch et al. [15] and Shumye et al. [16] found depression to be the strongest predictor of QoL across many sociodemographic and psychological factors, especially for mental health QoL, followed by environmental QoL, social QoL, and physical QoL.

Psychotherapy has proven effective in treating depression [17] and improving QoL [18]. A meta-analysis by Hofmann et al. [19] found a moderate effect of psychotherapy on QoL (Hedge’s g = .63). Similarly, Kolovos et al. [20] reported small to moderate effects, distinguishing between global QoL (g = 0.33, 95% CI 0.24–0.42), mental health (g = 0.42, 95% CI 0.33–0.51), and physical health (g = 0.16, 95% CI 0.05–0.27). However, access to psychotherapy is often limited due to financial, geographical, or provider shortages [21]. Left untreated, depression can further decrease QoL [19]. Internet-based interventions (IBIs), such as smartphone apps, offer a scalable, accessible, and cost-effective alternative to traditional psychotherapy. IBIs are digital tools designed to support individuals with various mental health conditions. Cognitive-behavioural therapy (CBT) is the most used approach in IBIs [22], as it includes techniques like cognitive restructuring, behavioural activation, and skill-building. Generally, CBT-based IBIs seem to outperform non-CBT IBIs [22]. The level of guidance in IBIs varies; guided IBIs are typically more effective than unguided ones [22], yet unguided IBIs are more scalable and accessible due to the lack of human interaction. Their effectiveness in treating depressive symptoms ranges from small [22–24] to medium effect sizes [25]. Meta-analyses by Firth et al. [26], Wu et al. [27], and Linardon et al. [22] found that IBIs significantly reduce depressive symptoms, though with small effect sizes (g = 0.28 in all analyses). A meta-analysis by Yang et al. [28] showed a moderate effect (g  = 0.72, 95% CI 0.479–0.962). Though the studies are similar in design, a minor difference between studies is that Yang et al. [28] focused on smartphone-based IBIs exclusively. In a systematic review by Hrynyschyn et al. [29], five out of eight studies found no significant difference between CBT IBIs and control groups regarding depression scores, while the remaining three reported small effect sizes.

Findings on the effect of IBIs on QoL remain inconsistent. A systematic review by Hrynyschyn et al. [29] indicated that studies found no significant improvement of QoL through IBIs. However, a more recent review by Fadipe et al. [30] suggested that internet-based cognitive-behavioural therapy (iCBT) can enhance QoL in individuals with depression, with the most pronounced benefits observed in women, younger individuals, patients with severe depression, and those with physical comorbidities. IBIs with some form of guidance generally outperformed unguided interventions. Maj et al. [31] reported a small effect of iCBT for depression on global QoL (g = 0.34, 95% CI 0.20–0.48) and a moderate effect on the physical health domain (g = 0.56, 95% CI 0.06–1.07). No significant differences were found between guided and unguided CBT-IBIs regarding global QoL, although the number of guided IBIs trials (n = 33) exceeded unguided IBIs trials (n = 9). Unlike the present meta-analysis, the authors only examined iCBT-IBIs and observed guided vs. unguided IBIs for depression and anxiety together. Similarly, Yang et al. [28] found a small but significant effect of IBIs on QoL (g = 0.25, 95% CI 0.064–0.457), without accounting for guidance levels in their analysis.

Beyond depression severity, the present meta-analysis focuses on the effect of unguided IBIs on QoL. While the aforementioned reviews have examined IBIs and QoL more broadly, they either did not distinguish between guided and unguided formats [28] or included mixed mental health conditions [31]. To our knowledge, this is the first meta-analysis to isolate unguided IBIs for depression only and to examine their effects not just on global QoL, but also across different QoL domains. We decided to focus on unguided IBIs since this format is particularly able to leverage the benefits of digital interventions such as low-threshold, instant availability.

The aim of this meta-analysis is to provide deeper insight into how effectively unguided IBIs improve QoL in adults between 18–65 years with depression. While previous meta-analyses have explored the link between IBIs and QoL in general, further research is needed to specifically examine the impact of unguided IBIs for depression on different QoL domains. This is—to the best of our knowledge—the first meta-analysis to research this topic. A multivariate, three-level meta-analysis was conducted, and pooled effect sizes were calculated.

## Methods

### Eligibility criteria

We registered this Meta-Analysis on the online platform PROSPERO (ID: CRD42023410012) on the 21st of March 2023.. The Preferred Reporting Items for Systematic Reviews and Meta-Analyses (PRISMA) checklist [32] was used in this meta-analysis. Participants had to be 18–65 years old. No cut-off score for depression was applied, allowing inclusion of participants without a clinical diagnosis. Studies including individuals with psychotic, bipolar, or acute suicidal conditions were excluded to reduce clinical heterogeneity. Only randomized controlled trials (RCTs) were included. Interventions had to be IBIs targeting depressive symptoms in an outpatient setting. Our meta-analysis focused on unguided IBIs, exclusively. Trials were excluded if researchers, psychologists, physicians, or trained professionals provided guidance concerning the use of the unguided IBI targeting depressive symptoms. Trials with additional face-to face or telemedicine interventions were also excluded. Control groups included waiting lists or treatment as usual (TAU). We labeled control conditions as TAU if participants had access to standard treatment common for the region of the study but were not granted access to the intervention after a waiting period. We labeled a control condition as waitlist if participants were able to access what we defined as TAU but were given access to the intervention after a waiting period. QoL assessment was mandatory as an outcome. Eligible studies were in English or German.

### Information sources

We searched the Embase Library, Cochrane Library, Medline and PsycInfo databases via the search platforms PubMed and OVID for articles published between the inception of the databases and the 1st of October 2024. Furthermore, grey literature was sought after in the Central Trial register of the Cochrane Library (CENTRAL) using the search string terms shown in the Appendix 3. Previous meta-analyses were individually investigated for eligible studies.

### Search strategy

A common search string for both data platforms was used, to have a comparable and standardized search method each time a search was conducted (see Appendix 3: Search String). Only RCTs performed in humans with an abstract in German or English were included. Duplicates were removed.

### Selection process

**Fig. 1:**
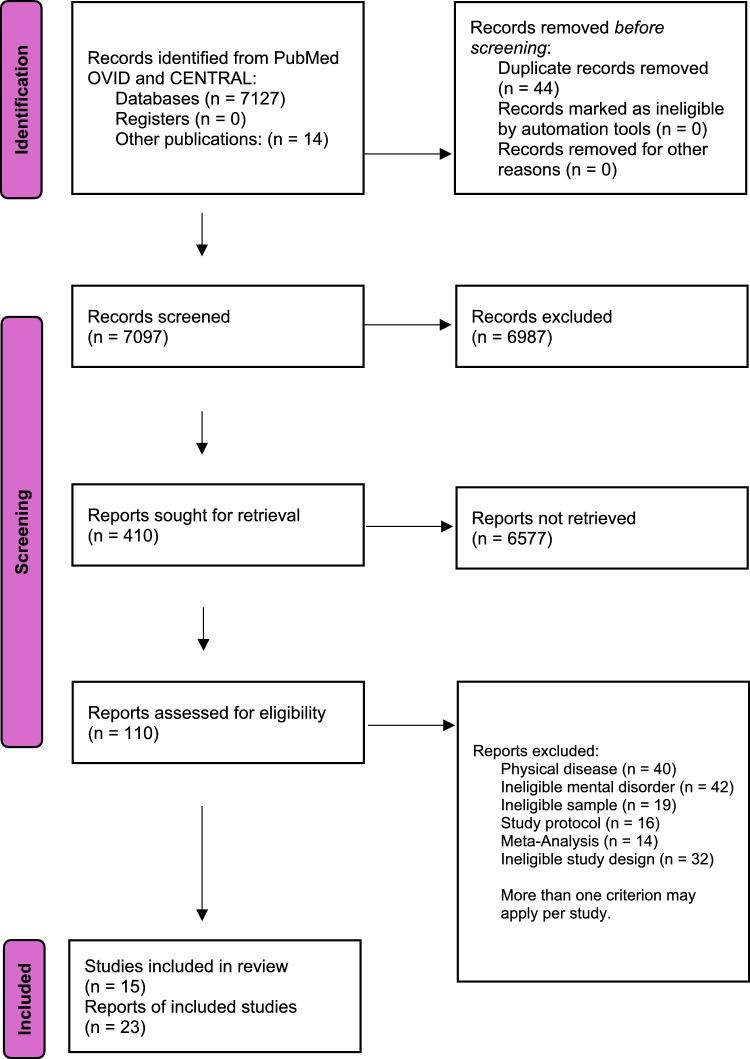
Flow chart of the selection process

Two researchers (R.H. and F.B.) independently screened each record, first by title and abstract, then by full-text evaluation based on inclusion and exclusion criteria. The process is shown in Fig. 1. *Rayyan*, an AI-based tool, was used to organize the selection process, detect duplicates, and keep decisions blinded until the selection was complete. If consensus could not be reached, a third expert was consulted (S.K.).

### Data items

We extracted data on global QoL, mental and physical health QoL subscales. This is a deviation from the registration on PROSPERO, where we did not specify subscales. We added the subscales because we expected the largest impact of IBIs to be on health related domains. Additionally, we thereby aimed to increase power since many trials only report health related QoL measures. Studies that reported neither were not included in the meta-analysis but were reviewed narratively. Additionally, data on depression severity, author, year of publication, type of control group, type of analysis (ITT, completer, per-protocol), inclusion criteria, sample size, number of treatments and control groups, measurement points, name, and type of intervention were extracted. To ensure accuracy, data extraction was performed independently by two reviewers (R.H, F.B.) and was then compared. All conflicts were resolved by discussion.

### Risk of bias assessment

The risk of bias assessment was conducted using the ROB-2 Tool (a revised Cochrane risk-of-bias tool for randomized trials, [33]). Bias was classified as low risk, some concerns, or high risk. Two independent researchers (R.H., F.B.) screened the studies, resolving conflicts through discussion. If consensus could not be reached, a third party was contacted. Bias estimates were based on selection bias, detection bias, attrition bias, bias in measurement of the outcome, and reporting bias. Attrition bias estimations were adjusted based on empirical data, a modification not pre-specified in our PROSPERO registration. This adjustment aimed to compare rate attrition bias within the context of typically observable attrition rates in IBI research. The average dropout rate for smartphone-based IBIs for depression is 26.2% (SD = 19.7%, 95% CI 18.12–36.34) [34]. Studies exceeding 45.9% dropout (mean + 1 SD) in the intervention group at post-measurement were classified as high risk, while those below 6.5% (mean−1 SD) were rated as low risk. Trials within this range were rated as "some concerns". All trials using appropriate subjective QoL measures were rated "some concerns", given QoL’s inherently subjective nature. Since all studies shared the same measurement bias, distinguishing between low and high risk was seen as unnecessary. The GRADE approach [35] was applied, classifying certainty of evidence as high, moderate, low, or very low based on risk of bias, inconsistency, indirectness, imprecision, and publication bias. For publication bias estimation, tests for small-study effect were performed, adjusted for multivariate analyses [36] and funnel-plots were generated. The aim of the funnel plots was to visually evaluate the existence of a publication bias in addition to statistical testing.

### Synthesis methods

All analyses were conducted in R (v4.2.3) using RStudio on Windows 10. We adopted a random-effects model due to assumed high heterogeneity. Heterogeneity was assessed using (multilevel) Higgins’ I^2^ and τ^2^, with the Hartung-Knapp adjustment enhancing robustness, and confidence intervals for τ^2^ and τ determined via the Q-profile method. A three-level multilevel meta-analysis was chosen over a two-level model given the clustered design of the interventions (many trials reported more than IBI). The decision for this model was based on an ANOVA, comparing AIC and BIC (prioritizing BIC for stricter model selection [37]). Effect sizes were reported as Hedges’ *g*. We interpreted g = 0.2, 0.5 and 0.8 as small, medium, and large effect sizes [38]. Influential studies were identified via Cook’s distance (a value > 1 was considered an outlier [38]). The data of one trial [39] was obtained through correspondence. Stuart et al. [40] did not provide standard deviations in their publication, which is why they were manually calculated by using the formula suggested by the Cochrane Handbook [41].

## Results

### Study characteristics

**Fig. 2:**
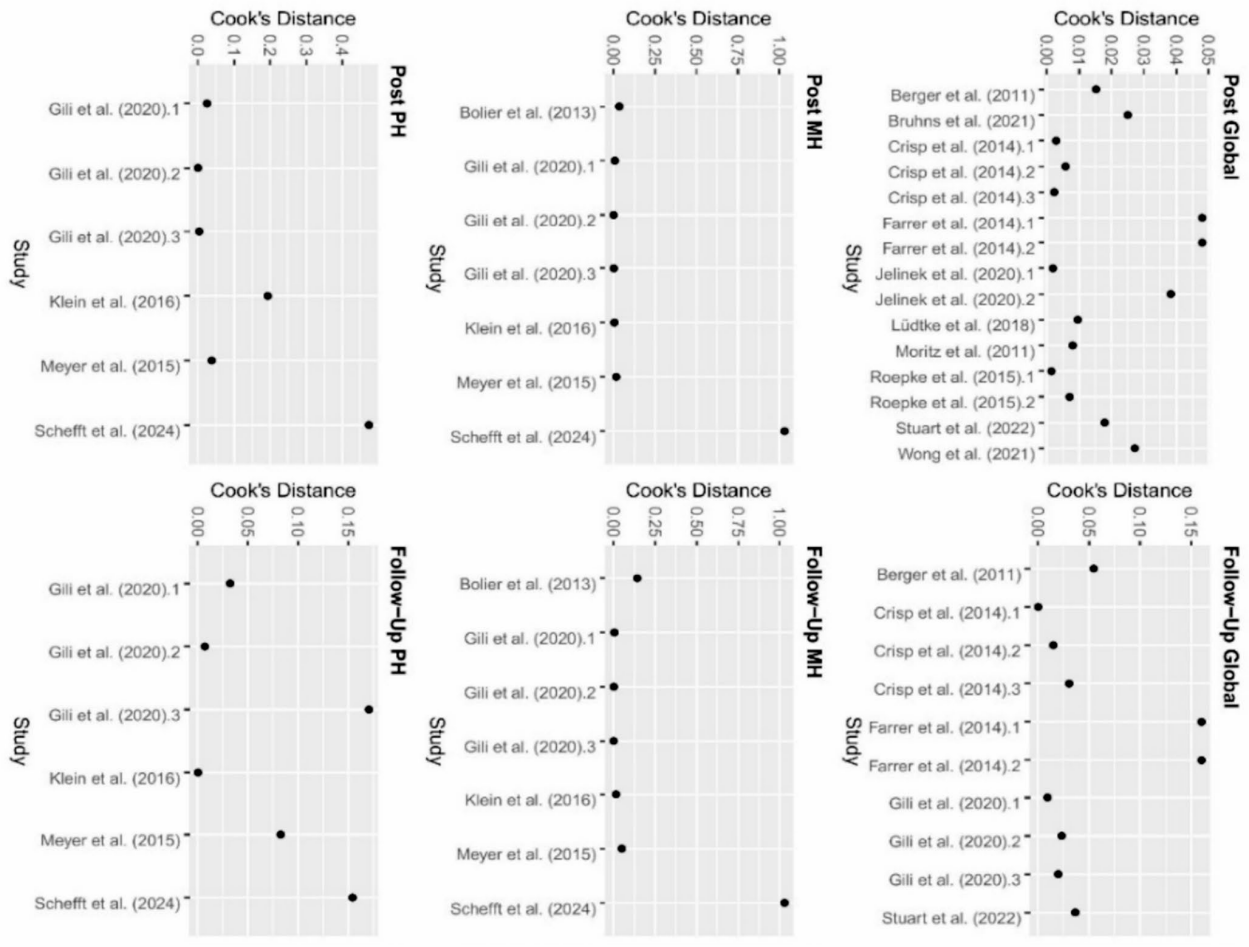
Cook’s distance of quality of life measurements. Global = global quality of life, MH = Mental health quality of life, PH = physical health quality of life

Starting with a total of n = 7097 studies, 15 eligible studies for the final analyses were found. The characteristics of the trials are presented in Table 1. Eight of the 15 studies were CBT-based, while others incorporated mindfulness, positive psychology, acceptance and commitment therapy, or health-focused elements (e.g., diet and physical activity). Studies primarily based on CBT were Berger et al. [42], Moritz et al. [43], Farrer et al. [44], Klein et al. [45], Lüdtke et al. [46], the iBA intervention in Jelinek et al. [47], Stuart et al. [40], and Schefft et al. [48]. Primarily non-CBT IBIs were the trials of Bolier et al. [49] , Crisp et al. [50], Roepke et al. [51], Meyer et al. [52], Gili et al. [39], Bruhns et al. [53], Wong et al. [54], and the mindfulness-based intervention in Jelinek et al. [47]. Intervention duration averaged 6.78 weeks (SD = 3.38), with a mean follow-up at 5.82 months, reported in 10 studies. The average sample size was 229.4 (SD = 129.32), totaling 3623 participants. Crisp et al. [51] had the largest sample (N = 478), while Berger et al. [42] had the smallest (N = 76). Two trials used waitlist controls, eleven used TAU, one "improved TAU" (general practitioner with extra training) [39], and one included both TAU and an active control with minimal support [47]. As shown in Fig. 2, no studies largely exceeded a Cook’s distance of 1 (Schefft et al. [48]: 1.03 at Post MH, 1.02 at follow-up MH) and therefore, no studies were identified as outliers.

**Table 1:** Descriptive data of the included studies

Authors/Date	Country	Intervention	Approach	Duration	Follow-up	Dep. score	QoL score	Sample size	Type of CG
Berger et al. [42]	Switz./ Germany	Deprexis	CBT	10 weeks	6 months	BDI-2	WHO-QoL	76 (25,25,26)	Waitlist
Jelinek et al. [47]	Germany	iBA	CBT, MF (AC)	2 weeks	1 month	PHQ-9	WHO-QoL	104 (37,32,35)	TAU
Gili et al. [39]	Spain	HLP, PAPP, MP	Health, PA, MF	12 weeks	6 & 12 months	PHQ-9	EQ-5D, SF-12*	221 (56,54,54,57)	iTAU
Lüdtke et al. [46]	Germany	Be Good To Yourself	CBT	4 weeks	n.a	PHQ-9	WHO-QoL	88 (44,44)	Waitlist
Bruhns et al. [53]	Germany	MCT & More	CBT, MF, ACT, MCT	4 weeks	n.a	PHQ-9	WHO-QoL	400 (200,200)	Waitlist
Stuart et al. [40]	USA	Thrive	CBT	8 weeks	6 months	PHQ-9	BRFSS	302 (148,154)	TAU
Wong et al. [54]	Hong K	Lifestyle Hub	TTM	9 weeks	n.a	BDI-2	SF-6D	79 (39,40)	Waitlist
Roepke et al. [51]	USA	SuperBetter	CBT, PS, SEA	ca. 4 weeks	6 weeks	CES-D	SWLS	283 (93,97,93)	Waitlist
Bolier et al. [49]	Netherl	PsyFit	PS	8 weeks	6 months	CES-D	MHC-SF, SF-36**	284 (143,141)	Waitlist
Klein et al. [45]	Germany	Deprexis	CBT	12 weeks	3 & 6 months	PHQ-9	SF-12	379 (187, 192)	TAU
Moritz et al. [43]	Germany	Deprexis	CBT	8 weeks	n.a	BDI-2	WHO-QoL	210 (105,105)	Waitlist
Meyer et al. [52]	Germany	Deprexis	CBT	12 weeks	6 months	PHQ-9	SF-12	163 (78, 85)	TAU
Crisp et al. [50]	Australia	ISG/ E-Couch	CBT and others	12 weeks	6 & 12 months	n.a	EUROHIS-QOL8	478 (82,85,89,78)	AC
Farrer et al. [44]	Australia	Mood Gym	CBT	6 weeks	6 & 12 months	n.a.***	EUROHIS-QOL8	155 (38,45,36,34)	TAU
Schefft et al. [48]	Germany	Selfapy	CBT	12 weeks	6 months	n.a	WHO-QoL	401 (151,150,100)	TAU

iBA, internet behavioural activation; HLP, psychoeducational program for the promotion of a healthy lifestyle; PAPP, Psychological intervention for the promotion of positive affect; CBT, cognitive behavioural therapy; MP, brief intervention based on mindfulness, MF, mindfulness, ACT, acceptance and commitment therapy; MCT, metacognitive therapy; PS,  positive psychology; TTM, transtheoretical model; SA, self-esteem and acceptance; BDI-2,  beck-depression inventory 2; WHO-QoL, World Health Organization QoL questionnaire; SWLS, satisfaction with life scale; BRFSS, behavioural risk factor surveillance; EQ-5D, European QoL 5 dimensions 3; SF-6D , short form six dimension, (i)TAU, treatment as usual. *only mental and physical scale ** only physical health and vitality scales ***no depression score but a psychological distress score was used

### Risk of bias

**Fig. 3:**
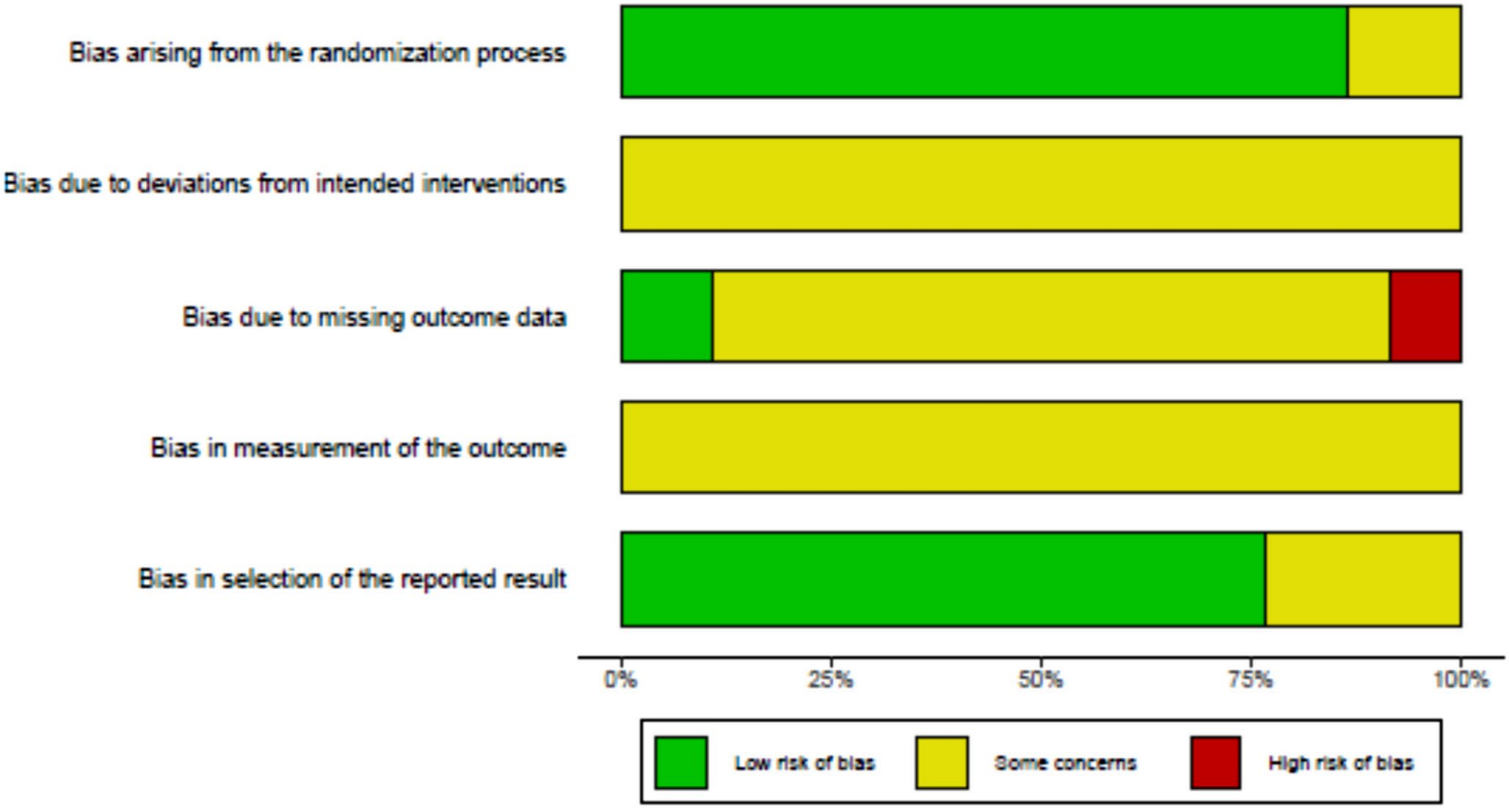
Summary of risk of bias assessment

**Fig. 4:**
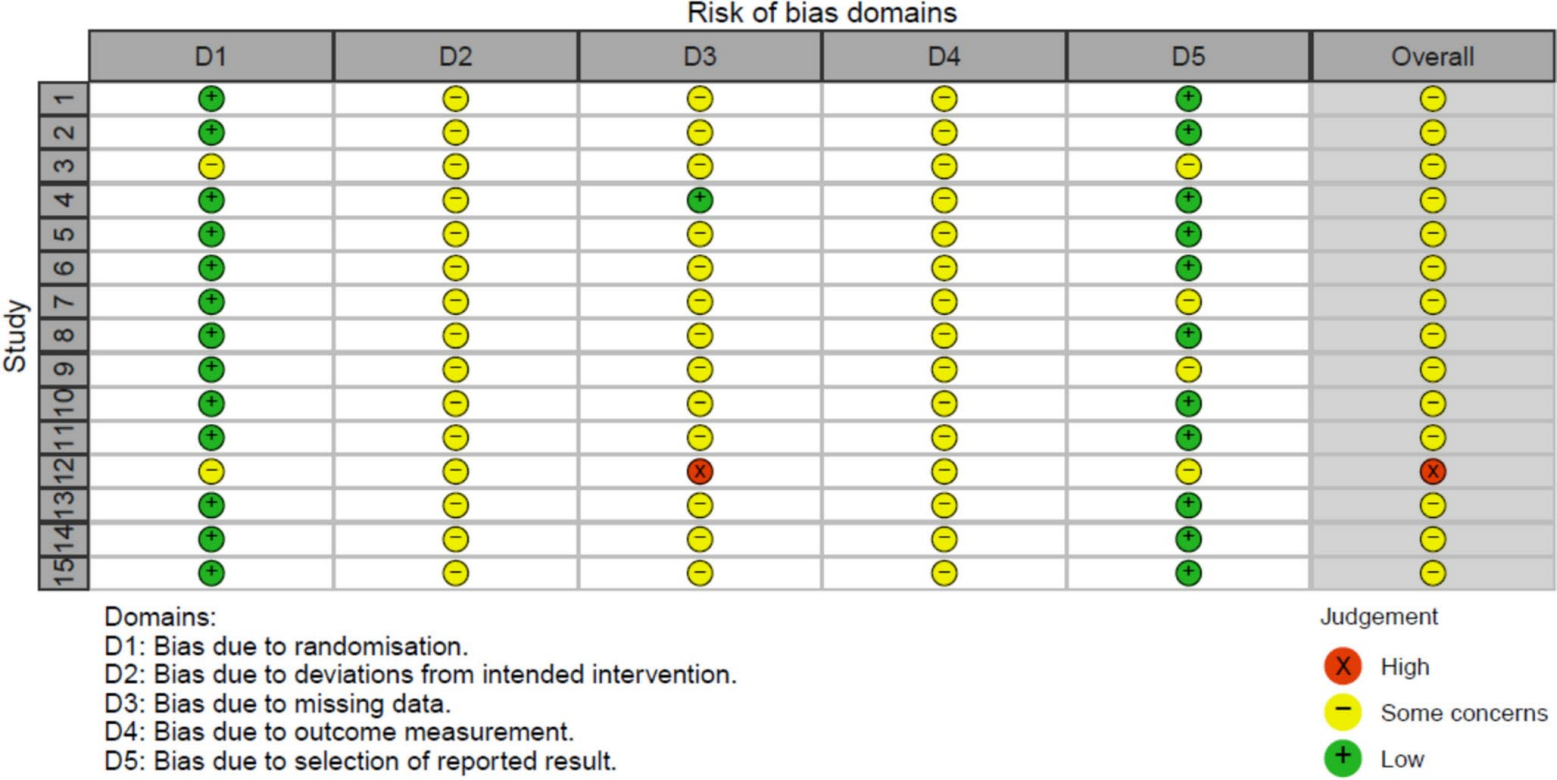
Traffic plot

The risk of bias assessment is shown in Figs. 3 and 4. While the usefulness of bias estimation regarding baseline differences in meta-analyses are debated [55], they are reported for completeness. Randomization was generally successful, with concealed allocation and no significant baseline differences, except in Roepke et al. [51] (age, gender) and Bruhns et al. [53] (sociodemographic factors of unclear relevance). IBIs in general are difficult to blind, so all studies showed some concern regarding deviation bias. Dropout rates were considerable, with a median of 27.62% at post-treatment and 41.93% at follow-up for QoL (Table 2). Most studies struggled with adherence issues, and many participants did not complete their IBIs. Crisp et al. [50] were rated low risk, while Roepke et al. [51] was rated high risk; all others were rated "some concerns"(bias due to missing data). Several studies [42, 47, 49–51] reported significant dropout differences between groups. Since only subjective QoL measures were used (Table 1), all studies received a "some concerns" rating (bias in measurement of the outcome). Thirteen of fifteen studies published a study protocol, but none was found for Lüdtke et al. [46] and Roepke et al. [51] (bias in selection of the reported result).

**Table 2:** Dropout rates of the eligible studies

Trials	Intervention post	Intervention follow-up	Control post	Control follow-up
Berger et al. [42]	12%	24%	15.38%	23.08%
Bolier et al. [49]	33.60%	37.8%	15.6%	22.7%
Bruhns et al. [53]	36%	n.a	32.5%	n.a
Crisp et al. [50]	4.1%	16.3–32.5%	7.79%	19.48–35.06%
Farrer et al. [44]	31%	41%	22.86%	37.14%
Gili et al. [39]	35.24%	49.83–51%	63.2%	64.9–70.2%
Jelinek et al. [47]	22%	28%	9%	11%
Klein et al. [45]	21.6%	24.6%	20.83%	25.4%
Lüdtke et al. [46]	16%	n.a	16%	n.a
Meyer et al. [52]	22%	27%	14%	28%
Moritz et al. [43]	18.1%	n.a	14.29%	n.a
Roepke et al. [51]	79.25%	87.89%	61.29%	68.82%
Schefft et al. [48]	22.67%	58.67%	48%	71%
Stuart et al. [40]	48%	57%	34%	24%
Wong et al. [54]	12.80%	n.a	12.5%	n.a
Mean	27.62%	41.89%	25.82%	36.91%
SD	18.17	20.14	18.2	21.58

The dropout rates refer to the number of patients that completed the QoL questionnaires within the targeted intervention group. If a study had multiple interventions, the numbers were averaged

#### Publication bias

**Fig. 5:**
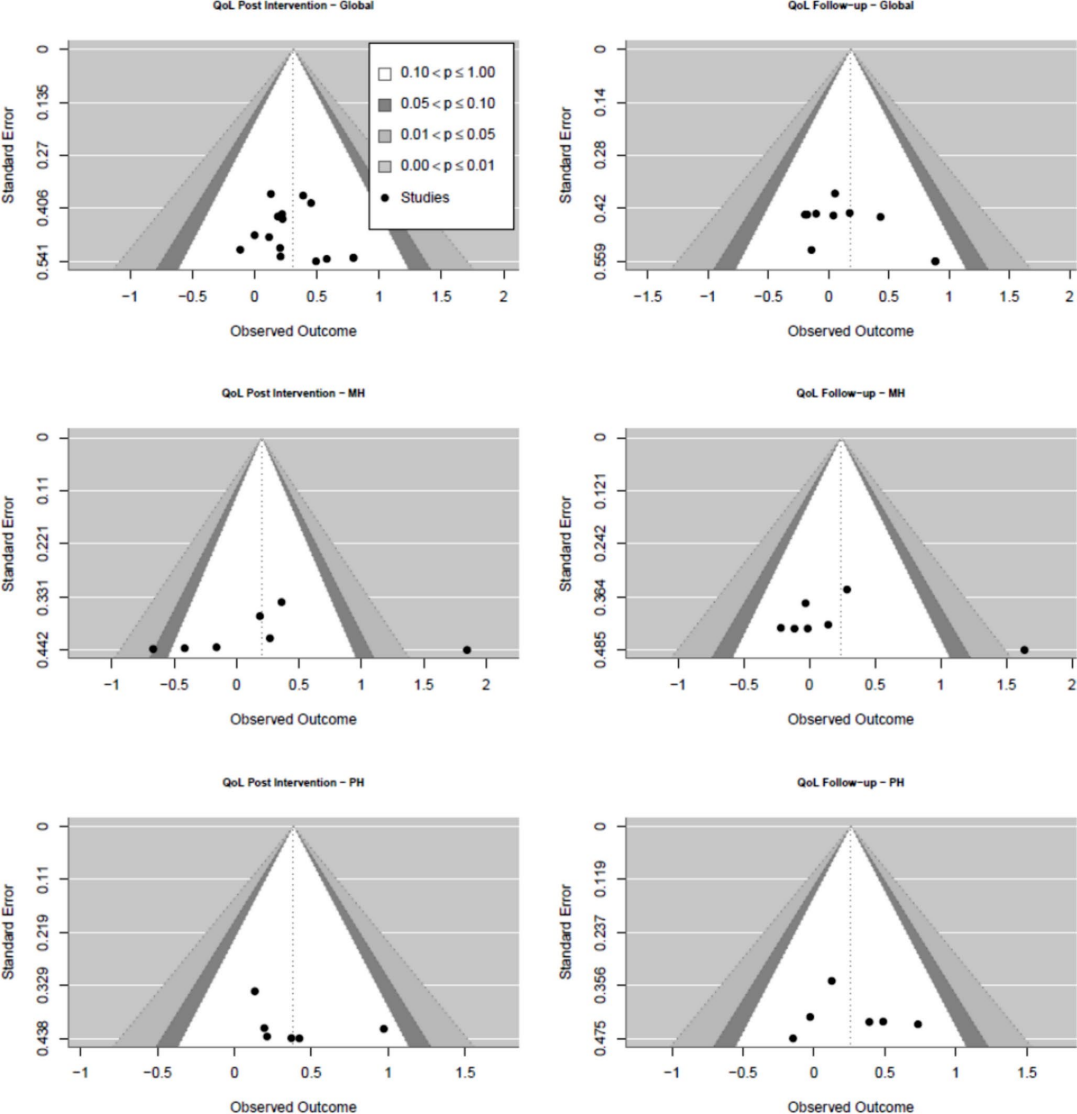
Funnel plot

**Fig. 6:**
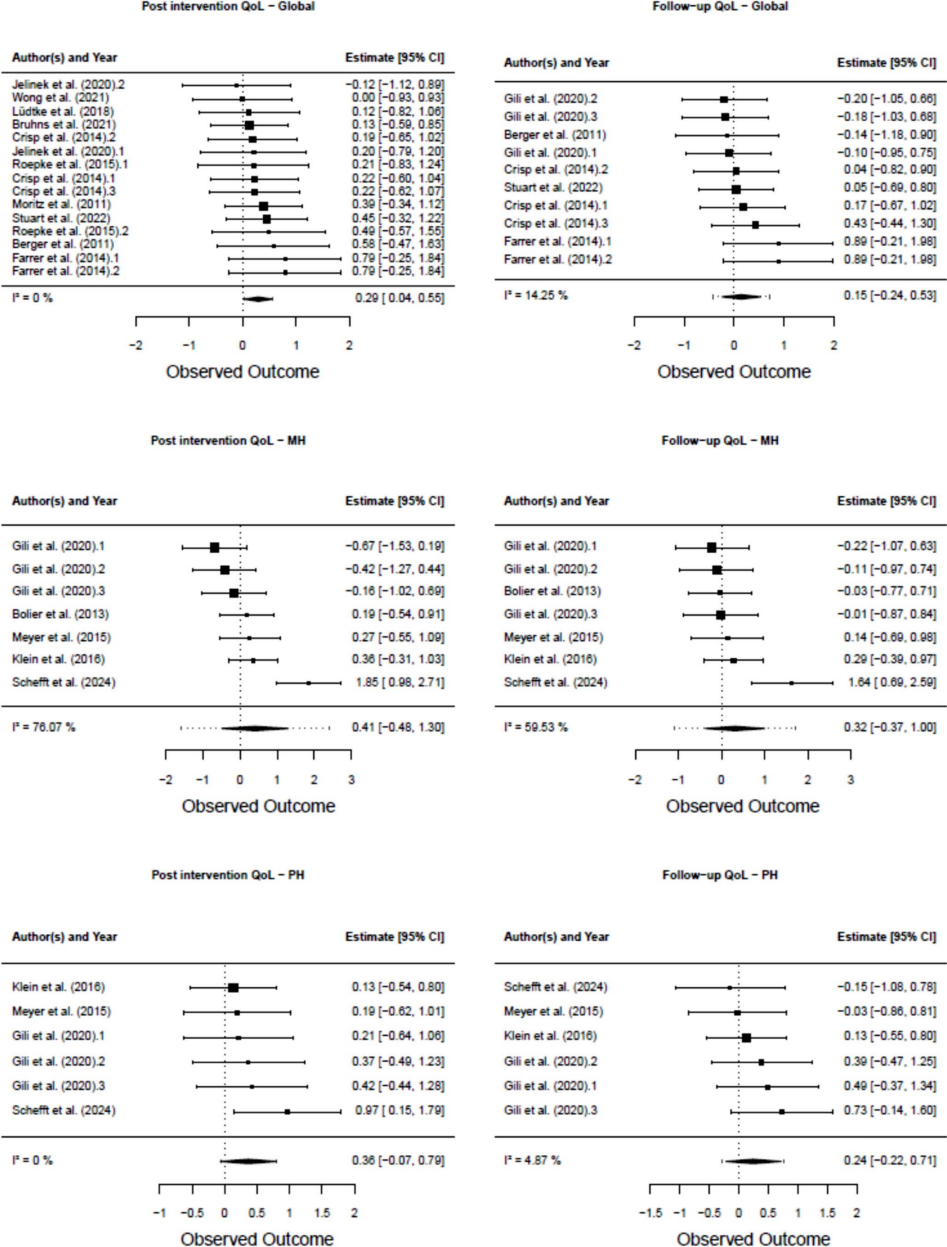
Forest-plots

For global QoL, there appears to be no visual identification of a publication bias (see Fig. 5), as well as for physical health QoL for both post and 6-month follow-up. The heterogeneity of results for mental health QoL is considerable (see Fig. 6), allowing for no precise statement about the publication bias in this domain. The test for small study effects was significant for the domains where heterogeneity was high: MH QoL at post and follow up both showed significant publication bias (both *p *< 0.01).

#### Results of statistical syntheses: quality of life

The results are presented in Table 3 and Fig. 6. A significant effect was only observed for global QoL at post intervention (g = 0.29, 95% CI 0.04–0.55, *p* = 0.03). All other analyses were not significant (*p*-values > 0.05). See the individual results in Appendix 1. In addition to the results of multivariate testing, there were two studies that reported one year follow-up [39, 50] and one study reporting 48 weeks follow up [45]. As previously mentioned, there were scores that did not fit into our categories (global, mental and physical QoL). Bolier et al. [49] showed a small but significant effect for the difference between their intervention and control group for the domain “Health” of the SF-36 at post (g = 0.33, 95% CI 0.05–0.60, *p* = 0.02), but no significant effect for said domain at follow-up (g = 0.01, 95% CI −0.27–0.29, *p* = 0.93). There was no significant effect for the “Vitality” domain of the SF-36 at post (g = 0.23, 95% CI −0.04–0.50, *p *= 0.10) and at 6-month follow-up (g = 0.04, 95% CI −0.24–0.32, *p* = 0.79). Schefft et al. [48] had a significant effect of the difference between intervention and control group for social relationships QoL at post (g = 0.69, 95% CI −0.35–1.03, *p *< 0.001).There was no significant effect for this domain at 6-month follow-up (g = 0.33, 95% CI −0.11–0.79, *p* = 0.13). For the QoL domain “Environment”, the effect was neither significant at post (g = 0.18, 95% CI−0.15–0.51, *p *= 0.31) nor 6-month follow-up (g = −0.32, 95% CI −0.77–0.13, *p* = 0.11).

**Table 3:** Results of quality of life syntheses

QoL measure	n	Effect size (g)	95% CI	*p*-value	Heterogeneity (I^2^)	95% PI
Global QoL (Post intervention)	13	0.29	0.04–0.55	0.03	0% (*p *= 0.98)	0.29 (0.04–0.55)
Mental health QoL (Post intervention)	7	0.41	−0.48–1.29	0.31	76.07% (*p *= 0.002)	0.39 (−0.45–0.86)
Physical health QoL (Post intervention)	6	0.36	0.07–0.79	0.08	0% (*p *= 0.90)	0.35 (−0.07–0.79)
Global QoL (6-Month follow-up)	10	0.15	−0.24–0.53	0.75	14.25% (*p *= 0.96)	0.15 (−0.24–0.49)
Mental health QoL (6-Month follow-up)	5	0.32	−0.37–1.00	0.09	59.33% (*p *= 0.052)	0.33 (−0.42–0.82)
Physical health QoL (6-Month follow-up)	6	0.24	−0.22–0.70	0.72	4.87% (*p *= 0.72)	0.24 (−0.22–0.61)

#### Secondary measure: depression

The pooled effect was significant (g = − 0.37 (95% CI −0.62 to −0.12, *p *= 0.01) and can be considered small. The prediction value was −0.35 (95% PI: − 0.55 to − 0.12). For 6-month follow-up, the pooled effect was not significant (g = −0.26 (95% CI −0.60–0.08. *p *= 0.12). The prediction value was −0.25 (95% PI: − 0.54–0.08). The heterogeneity was I^2 ^= 0%.

## Discussion

In this meta-analysis, we examined the effect of unguided IBIs for depression on QoL. A small but significant improvement in global QoL at post-treatment was found compared to control groups, but no significant differences emerged for mental and physical health QoL at post-treatment or follow-up. The observed effect on global QoL (Hedges’ g = 0.29) represents a small to moderate standardized mean difference. While this corresponds to a change of 0.29 SD, effects of comparable magnitude have been shown to translate into clinically meaningful differences on established QoL measures, which is approximately 8–10 points on the WHOQOL-BREF [56], 0.05–0.10 on the EQ-5D [57], 3–5 points on the SF-36 or SF-12 summary scores [58], and 2–4 points on the SWLS [59]. These ranges are commonly considered minimally clinically important differences, suggesting that even relatively small effects may hold practical significance for patients. A secondary analysis showed a short-term improvement in depressive symptoms, but this effect did not persist beyond six months.

This is the first meta-analysis focusing on unguided IBIs for depression and QoL. Prior research that did not differentiate between guided and unguided IBIs reported similar small effects [28, 30, 31]. However, inclusion criteria in other reviews deviated from our analysis limiting comparability. Fadipe et al. [30] included patients from various clinical settings, including inpatient and outpatient settings, which increases heterogeneity. Maj et al. [31] pooled iCBT studies for both depression and anxiety, with only five of 39 studies focusing on unguided IBIs for depression. Unlike previous research, we included subthreshold depression, which possibly broadens the applicability.

Several mechanisms through which unguided IBIs affect QoL can be discussed. QoL improvements may occur indirectly through symptom reduction, as lower symptom burden is linked to better QoL [55]. Some interventions may also target specific QoL domains more directly. For example, behavioral activation can promote activity and structure, supporting physical QoL [55]. The observed effects must be interpreted in light of the substantial variability across interventions, target groups, and QoL measures. Rather than reflecting a uniform effect of unguided IBIs, the results likely capture the diversity of approaches and mechanisms used across studies. For instance, both Stuart et al. [40] and Berger et al. [42] investigated primarily CBT-based IBIs but used different approaches. Stuart et al. [40] analyzed a 10-lesson video course focusing on behavioral activation, cognitive restructuring, and social skills training, whereas Berger et al. [42] also included psychoeducation, relaxation, and mindfulness. In general, CBT-based interventions showed superior QoL improvements compared to non-CBT IBIs [22].

The findings on depression scores align with prior research. Unguided IBIs show small to moderate effects on depressive symptoms [22–24]. Meta-analyses by Cuijpers et al. [60] and Linardon et al. [22] indicate that unguided IBIs are generally less effective than guided self-help. However, more recent trials support their effectiveness in symptom reduction [48, 61].

Depression and QoL are inherently linked [3, 4], suggesting that reducing depressive symptoms should improve QoL. Our findings hint in that direction but highlight a complex relationship. In the present dataset, global QoL was assessed in 15 comparisons, compared to 7 comparisons for mental health and 6 for physical health. The higher number of comparisons and larger total sample likely increased statistical power. Moreover, global QoL measures often include more items and span multiple domains, which can enhance reliability and reduce measurement error. In contrast, subscales like mental and physical QoL are typically based on fewer items, which may reduce sensitivity to change. This could help explain why only global QoL showed a significant effect. Maj et al. [31] reported that IBIs improved physical QoL more than psychological, social, or environmental domains. This is an unexpected pattern, given that depression typically affects psychological QoL most strongly. This was not replicated in the present meta-analysis. Maj et al. [31] suggested that small sample sizes may have limited the generalizability of domain-specific effects. In a single trial, Schefft et al. [48] found improvements in both psychological and physical QoL, with only psychological benefits persisting at follow-up. These mixed findings point to the need for a more differentiated understanding of how unguided IBIs influence specific QoL domains. Depression is most strongly associated with impairments in psychological QoL, followed by environmental and social domains, while physical QoL tends to be less affected [15, 16]. Future research should examine whether IBIs that align more closely with these patterns produce more consistent effects. When developing IBIs, it is essential to consider how depression differentially impacts specific QoL subdomains, allowing interventions to target the most severely impaired areas more effectively. For example, if meta-analyses show that the apps have a pronounced effect on mental QoL but not on physical, modules could be added that address physical well-being in both general and personalized ways (exercise, nutritional choices, dealing with physical pain or neglect of chronic health conditions due to depression).

Despite some small studies and moderate publication bias in the mental health QoL subdomain, the overall certainty of evidence was rated as moderate to high, given the low heterogeneity across most studies, the similarity in populations and measurement tools, and the high precision of findings, supported by exceeding the optimal information size of 385 and overlapping confidence intervals.

### Limitations

A key limitation of this meta-analysis is the small number of included studies, limiting the detection of small effects. Additional limitations stem from trial design. Considerable dropout rates and low adherence raise concerns, and most studies did not report reasons for dropout. Furthermore, relying on self-reported QoL measures introduces potential bias. While subjective assessments are standard in web-based research, they lack objective validation. Recent research suggests that QoL is shaped not only by objective factors (e.g., healthcare access, housing) but also by subjective experiences like relationship quality and personal fulfillment [62].

### Implications for practice and research

For general practice, unguided IBIs can be used to treat depressive symptoms and improve QoL, but the observed effects were small and short-term. These findings support the use of unguided IBIs as a low-intensity first step within a stepped-care model. Regular monitoring can identify those who require more intensive interventions. Yet, given the low sample size, conclusions should be drawn cautiously. Regarding research, to enhance the effectiveness of unguided IBIs, it is crucial to determine which intervention components provide the most benefit. Component analyses could improve IBIs beyond symptom reduction. Future research should explore ways to improve adherence and reduce dropout rates.

## Data Availability

The data we used for analysis will not be published online but can be sent upon request.

## Appendix 1

See Table 4.

**Table 4:** Raw and standardized quality of life data

Study	Time	Measure	Comparison	Questionnaire	QoL.scale	IG			CG			p	Pooled SD	g	S.E.	CI−	CI +
						Mean	n	SD	Mean	n	SD						
Berger et al. [42]		pre	IG1 vs CG	WHO-QoL	Global	41.5	25	15.6	38.9	26	18.1	0.58	16.92	0.15	0.28	−0.41	0.72
Berger et al. [42]	10 weeks	post T1	IG1 vs CG	WHO-QoL	Global	52	25	20.9	40.8	26	17.1	0.04	19.06	0.58	0.29	0.01	1.16
Berger et al. [42]	24 weeks	post T2	IG vs. CG	WHO-QoL	Global	51.9	25	22.9	55.2	26	22.8	0.61	22.85	−0.14	0.28	−0.71	0.42
Bolier et al. [49]		pre	IG vs CG	SF-36	Health	18.57	143	3.51	17.79	141	3.66	0.07	3.59	0.22	0.12	−0.02	0.45
Bolier et al. [49]		pre	IG vs CG	SF-36	Vitality	14.67	143	3.17	14.66	141	2.82	0.98	3.00	0.00	0.12	−0.23	0.24
Bolier et al. [49]		pre	IG vs CG	MHC-SF	MH	3.52	143	0.63	3.51	141	0.63	0.89	0.63	0.02	0.12	−0.22	0.25
Bolier et al. [49]	8 weeks	post T1	IG vs CG	SF-36	Health	19.21	95	3.16	18.09	119	3.61	0.02	3.42	0.33	0.14	0.05	0.60
Bolier et al. [49]	8 weeks	post T1	IG vs CG	SF-36	Vitality	15.8	95	3.39	15.09	119	2.86	0.10	3.11	0.23	0.14	−0.04	0.50
Bolier et al. [49]	8 weeks	post T1	IG vs CG	MHC-SF	MH	3.95	95	0.59	3.81	119	0.85	0.16	0.75	0.19	0.14	−0.08	0.46
Bolier et al. [49]	24 weeks	post T2	IG vs CG	SF-36	Health	18.65	89	3.41	18.61	109	3.36	0.93	3.38	0.01	0.14	−0.27	0.29
Bolier et al. [49]	24 weeks	post T2	IG vs CG	SF-36	Vitality	15.53	89	3.99	15.39	109	3.15	0.79	3.55	0.04	0.14	−0.24	0.32
Bolier et al. [49]	24 weeks	post T2	IG vs CG	MHC-SF	MH	3.89	89	0.52	3.91	109	0.76	0.83	0.66	−0.03	0.14	−0.31	0.25
Bruhns et al. [53]		pre	IG vs. CG	WHO-QoL	Global	3.74	90	0.8	3.67	135	0.76	0.51	0.78	0.09	0.14	−0.18	0.36
Bruhns et al. [53]	4 weeks	post T1	IG vs. CG	WHO-QoL	Global	3.86	90	0.82	3.76	135	0.72	0.35	0.76	0.13	0.14	−0.14	0.40
Crisp et al. [50]		pre	E-Couch vs. CG	Eurohis-QOL8	Global	3.28	74	0.67	3.2	74	0.68	0.47	0.68	0.12	0.16	−0.21	0.44
Crisp et al. [50]	12 weeks	post T1	E-Couch vs. CG	Eurohis-QOL8	Global	3.58	59	0.81	3.41	71	0.73	0.22	0.77	0.22	0.18	−0.13	0.57
Crisp et al. [50]	24 weeks	post T2	E-Couch vs. CG	Eurohis-QOL8	Global	3.52	54	0.77	3.39	62	0.71	0.35	0.74	0.17	0.19	−0.19	0.55
Crisp et al. [50]	1 year	post T3	E-Couch vs. CG	Eurohis-QOL8	Global	3.57	46	0.75	3.44	52	0.75	0.39	0.75	0.17	0.20	−0.23	0.58
Crisp et al. [50]		pre	ISP vs. CG	Eurohis-QOL8	Global	3.23	77	0.71	3.2	74	0.68	0.79	0.70	0.04	0.16	−0.28	0.36
Crisp et al. [50]	12 weeks	post T1	ISP vs. CG	Eurohis-QOL8	Global	3.55	53	0.77	3.41	71	0.73	0.31	0.75	0.19	0.18	−0.17	0.55
Crisp et al. [50]	24 weeks	post T2	ISP vs. CG	Eurohis-QOL8	Global	3.42	48	0.79	3.39	62	0.71	0.84	0.75	0.04	0.19	−0.34	0.42
Crisp et al. [50]	1 year	post T3	ISP vs. CG	Eurohis-QOL8	Global	3.5	42	0.86	3.44	52	0.75	0.72	0.80	0.07	0.21	−0.34	0.49
Crisp et al. [50]		Pre	ISP+E-Couch vs. CG	Eurohis-QOL8	Global	3.32	73	0.7	3.2	74	0.68	0.29	0.69	0.17	0.17	−0.15	0.50
Crisp et al. [50]	12 weeks	post T1	ISP+E-Couch vs. CG	Eurohis-QOL8	Global	3.58	48	0.79	3.41	71	0.73	0.24	0.75	0.22	0.19	−0.15	0.60
Crisp et al. [50]	24 weeks	post T2	ISP+E-Couch vs. CG	Eurohis-QOL8	Global	3.7	47	0.72	3.39	62	0.71	0.03	0.71	0.43	0.20	0.05	0.82
Crisp et al. [50]	1 year	post T3	ISP+E-Couch vs. CG	Eurohis-QOL8	Global	3.72	36	0.74	3.44	52	0.75	0.09	0.75	0.37	0.22	−0.06	0.81
Farrer et al. (2014)		Pre	Web only vs. CG	Eurohis-QOL8	Global	12.24	38	5.72	12.06	35	4.81	0.88	5.30	0.03	0.23	−0.43	0.50
Farrer et al. (2014)	6 weeks	post T1	Web only vs. CG	Eurohis-QOL8	Global	16.7	27	6.83	11.56	27	5.91	0.00	6.39	0.79	0.28	0.24	1.37
Farrer et al. (2014)	24 weeks	post T2	Web only vs. CG	Eurohis-QOL8	Global	17.3	23	7.38	11.14	22	6.2	0.00	6.83	0.89	0.31	0.27	1.53
Farrer et al. (2014)	6 weeks	post T1	Web only vs. CG	Eurohis-QOL8	Global	16.7	27	6.83	11.56	27	5.91	0.00	6.39	0.79	0.28	0.24	1.37
Farrer et al. (2014)	24 weeks	post T2	Web only vs. CG	Eurohis-QOL8	Global	17.3	23	7.38	11.14	22	6.2	0.00	6.83	0.89	0.31	0.27	1.53
Gili et al. [39]		pre	IG (PAPP) vs. CG (iTAU)	EQ-5D	Global	47.86	56	20.33	51.75	57	21.72	0.33	21.04	−0.18	0.19	−0.56	0.19
Gili et al. [39]		pre	IG (PAPP) vs. CG (iTAU)	SF-12	PH	42.45	56	9.83	42.16	57	10.66	0.88	10.26	0.03	0.19	−0.34	0.40
Gili et al. [39]		pre	IG (PAPP) vs. CG (iTAU)	SF-12	MH	26.22	56	8.08	27.59	57	9.61	0.41	8.88	−0.15	0.19	−0.53	0.22
Gili et al. [39]		pre	IG (HLP) vs. CG (iTAU)	EQ-5D	Global	52.22	54	21.78	51.75	57	21.72	0.91	21.75	0.02	0.19	−0.35	0.40
Gili et al. [39]		pre	IG (HLP) vs. CG (iTAU)	SF-12	PH	42.52	54	9.75	42.16	57	10.66	0.85	10.23	0.03	0.19	−0.34	0.41
Gili et al. [39]		pre	IG (HLP) vs. CG (iTAU)	SF-12	MH	29.96	54	10.86	27.59	57	9.61	0.23	10.24	0.23	0.19	−0.15	0.61
Gili et al. [39]		pre	IG (MP) vs. CG (iTAU)	EQ-5D	Global	47.59	54	25.69	51.75	57	21.72	0.36	23.73	−0.17	0.19	−0.55	0.20
Gili et al. [39]		pre	IG (MP) vs. CG (iTAU)	SF-12	PH	45.17	54	13.54	42.16	57	10.66	0.20	12.15	0.25	0.19	−0.13	0.63
Gili et al. [39]		pre	IG (MP) vs. CG (iTAU)	SF-12	MH	26.22	54	9.97	27.59	57	9.61	0.46	9.79	−0.14	0.19	−0.52	0.24
Gili et al. [39]	12 weeks	post T1	IG (PAPP) vs. CG (iTAU)	SF-12	PH	45.11	56	11.32	42.57	57	12.47	0.26	11.91	0.21	0.19	−0.16	0.59
Gili et al. [39]	12 weeks	post T1	IG (PAPP) vs. CG (iTAU)	SF-12	MH	30.5	56	10.43	38.22	57	12.39	0.00	11.46	−0.67	0.19	−1.06	−0.29
Gili et al. [39]	12 weeks	post T1	IG (HLP) vs. CG (iTAU)	SF-12	PH	47.15	54	12.09	42.57	57	12.47	0.05	12.29	0.37	0.19	−0.01	0.75
Gili et al. [39]	12 weeks	post T1	IG (HLP) vs. CG (iTAU)	SF-12	MH	32.9	54	13.01	38.22	57	12.39	0.03	12.70	−0.42	0.19	−0.80	−0.04
Gili et al. [39]	12 weeks	post T1	IG (MP) vs. CG (iTAU)	SF-12	PH	47.88	54	12.54	42.57	57	12.47	0.03	12.50	0.42	0.19	0.04	0.81
Gili et al. [39]	12 weeks	post T1	IG (MP) vs. CG (iTAU)	SF-12	MH	36.15	54	13.17	38.22	57	12.39	0.40	12.78	−0.16	0.19	−0.54	0.21
Gili et al. [39]	24 weeks	post T2	IG (PAPP) vs. CG (iTAU)	EQ-5D	Global	62.68	56	18.24	64.63	57	18.95	0.58	18.60	−0.10	0.19	−0.48	0.27
Gili et al. [39]	24 weeks	post T2	IG (PAPP) vs. CG (iTAU)	SF-12	PH	45.48	56	10.15	40.09	57	11.74	0.01	10.98	0.49	0.19	0.11	0.87
Gili et al. [39]	24 weeks	post T2	IG (PAPP) vs. CG (iTAU)	SF-12	MH	38.61	56	10.83	41.22	57	12.86	0.25	11.90	−0.22	0.19	−0.59	0.15
Gili et al. [39]	24 weeks	post T2	IG (HLP) vs. CG (iTAU)	EQ-5D	Global	60.81	54	19.69	64.63	57	18.95	0.30	19.31	−0.20	0.19	−0.58	0.18
Gili et al. [39]	24 weeks	post T2	IG (HLP) vs. CG (iTAU)	SF-12	PH	44.82	54	12.35	40.09	57	11.74	0.04	12.04	0.39	0.19	0.01	0.77
Gili et al. [39]	24 weeks	post T2	IG (HLP) vs. CG (iTAU)	SF-12	MH	39.69	54	13.65	41.22	57	12.86	0.55	13.25	−0.11	0.19	−0.49	0.26
Gili et al. [39]	24 weeks	post T2	IG (MP) vs. CG (iTAU)	EQ-5D	Global	61	54	21.6	64.63	57	18.95	0.35	20.28	−0.18	0.19	−0.56	0.20
Gili et al. [39]	24 weeks	post T2	IG (MP) vs. CG (iTAU)	SF-12	PH	48.11	54	9.87	40.09	57	11.74	0.00	10.87	0.73	0.20	0.35	1.13
Gili et al. [39]	24 weeks	post T2	IG (MP) vs. CG (iTAU)	SF-12	MH	41.04	54	13.44	41.22	57	12.86	0.94	13.15	−0.01	0.19	−0.39	0.36
Gili et al. [39]	1 year	post T3	IG (PAPP) vs. CG (iTAU)	EQ-5D	Global	66.57	56	19.04	68.33	57	19.28	0.63	19.16	−0.09	0.19	−0.46	0.28
Gili et al. [39]	1 year	post T3	IG (PAPP) vs. CG (iTAU)	SF-12	PH	47.04	56	10.66	47.53	57	8.69	0.79	9.72	−0.05	0.19	−0.42	0.32
Gili et al. [39]	1 year	post T3	IG (PAPP) vs. CG (iTAU)	SF-12	MH	35.26	56	13.42	38.46	57	13.02	0.20	13.22	−0.24	0.19	−0.62	0.13
Gili et al. [39]	1 year	post T3	IG (HLP) vs. CG (iTAU)	EQ-5D	Global	61.78	54	20.34	68.33	57	19.28	0.08	19.80	−0.33	0.19	−0.71	0.05
Gili et al. [39]	1 year	post T3	IG (HLP) vs. CG (iTAU)	SF-12	PH	45.68	54	11.08	47.53	57	8.69	0.33	9.92	−0.19	0.19	−0.56	0.19
Gili et al. [39]	1 year	post T3	IG (HLP) vs. CG (iTAU)	SF-12	MH	38.4	54	13.43	38.46	57	13.02	0.98	13.22	0.00	0.19	−0.38	0.37
Gili et al. [39]	1 year	post T3	IG (MP) vs. CG (iTAU)	EQ-5D	Global	65.17	54	19.44	68.33	57	19.28	0.39	19.36	−0.16	0.19	−0.54	0.21
Gili et al. [39]	1 year	post T3	IG (MP) vs. CG (iTAU)	SF-12	PH	48.02	54	10.69	47.53	57	8.69	0.79	9.71	0.05	0.19	−0.33	0.43
Gili et al. [39]	1 year	post T3	IG (MP) vs. CG (iTAU)	SF-12	MH	39.08	54	13.39	38.46	57	13.02	0.81	13.20	0.05	0.19	−0.33	0.42
Jelinek et al. [47]		pre	iBA vs CG	WHO-QoL	Global	3.08	37	0.68	2.94	35	0.73	0.40	0.70	0.20	0.24	−0.27	0.67
Jelinek et al. [47]		pre	MF vs CG	WHO-QoL	Global	2.91	32	0.86	2.94	35	0.73	0.88	0.79	−0.04	0.24	−0.53	0.45
Jelinek et al. [47]	2 weeks	post T1	iBA vs CG	WHO-QoL	Global	3.28	29	0.88	3.12	32	0.66	0.43	0.77	0.20	0.26	−0.31	0.72
Jelinek et al. [47]	2 weeks	post T1	MF vs CG	WHO-QoL	Global	3.04	27	0.71	3.12	32	0.66	0.66	0.68	−0.12	0.26	−0.64	0.41
Jelinek et al. [47]	4 weeks	post T2	iBA vs CG	WHO-QoL	Global	3.11	27	1.01	2.94	31	0.77	0.48	0.89	0.19	0.26	−0.34	0.72
Jelinek et al. [47]	4 weeks	post T2	MF vs CG	WHO-QoL	Global	3.05	21	0.74	2.94	31	0.77	0.61	0.76	0.14	0.28	−0.42	0.71
Klein et al. [45]		pre	IG vs. CG	SF-12	PH	48.61	183	9.10	47.9	184	9.43	0.46	9.27	0.08	0.10	−0.13	0.28
Klein et al. [45]	12 weeks	post T1	IG vs CG	SF-12	PH	48.80	147	9.24	47.56	150	9.46	0.25	9.35	0.13	0.12	−0.10	0.36
Klein et al. [45]	24 weeks	post T2	IG vs CG	SF-12	PH	48.20	143	9.69	46.97	137	9.88	0.29	9.78	0.13	0.12	−0.11	0.36
Klein et al. [45]	48 weeks	post T3	IG vs CG	SF-12	PH	49.10	123	9.30	48.08	124	9.53	0.40	9.42	0.11	0.13	−0.14	0.36
Klein et al. [45]		pre	IG vs. CG	SF-12	MH	34.63	183	7.99	34.39	184	8.14	0.78	8.07	0.03	0.10	−0.18	0.24
Klein et al. [45]	12 weeks	post T1	IG vs CG	SF-12	MH	41.97	147	10.65	38.16	150	10.43	0.00	10.54	0.36	0.12	0.13	0.59
Klein et al. [45]	24 weeks	post T2	IG vs CG	SF-12	MH	43.01	143	11.51	39.67	137	11.8	0.02	11.65	0.29	0.12	0.05	0.52
Klein et al. [45]	48 weeks	post T3	IG vs CG	SF-12	MH	43.62	123	11.07	41.6	124	11	0.15	11.04	0.18	0.13	−0.07	0.43
Lüdtke et al. [46]		pre	IG vs. CG	WHO-QoL	Global	79.4	44	12.85	75.32	44	14.55	0.17	13.73	0.29	0.21	−0.13	0.72
Lüdtke et al. [46]	4 weeks	post T1	IG vs. CG	WHO-QoL	Global	80.76	37	13.29	79.05	39	15.76	0.61	14.61	0.12	0.23	−0.34	0.57
Meyer et al. [52]		pre	IG vs. CG	SF-12	PH	45.39	78	10.72	43.79	85	10.45	0.34	10.58	0.15	0.16	−0.16	0.46
Meyer et al. [52]	12 weeks	post T1	IG vs CG	SF-12	PH	45.82	61	9.45	43.74	73	11.58	0.25	10.66	0.19	0.17	−0.15	0.54
Meyer et al. [52]	24 weeks	post T2	IG vs CG	SF-12	PH	41.6	57	9.14	41.88	64	11.36	0.88	10.37	−0.03	0.18	−0.39	0.33
Meyer et al. [52]		pre	IG vs. CG	SF-12	MH	25.93	78	6.95	24.65	85	7.31	0.25	7.14	0.18	0.16	−0.13	0.49
Meyer et al. [52]	12 weeks	post T1	IG vs CG	SF-12	MH	33.7	61	11.24	30.62	73	11.61	0.12	11.44	0.27	0.17	−0.08	0.61
Meyer et al. [52]	24 weeks	post T2	IG vs CG	SF-12	MH	34.58	57	11.74	32.89	64	11.82	0.43	11.78	0.14	0.18	−0.22	0.50
Moritz et al. (2011)		pre	IG vs CG	WHOO-QoL	Global	67.13	105	12.93	66.99	105	11.46	0.93	12.22	0.01	0.14	−0.26	0.28
Moritz et al. (2011)	8 weeks	post T1	IG vs CG	WHOO-QoL	Global	73.42	105	14.27	68.26	105	12.04	0.01	13.20	0.39	0.14	0.12	0.67
Roepke et al. [51]		pre	IG1(CBT-PPT SB) vs. CG	SWLS	Global	13.2	93	6.04	14.17	93	6.07	0.28	6.06	−0.16	0.15	−0.45	0.13
Roepke et al. [51]		pre	IG2 (SB) vs CG	SWLS	Global	14.12	97	6.38	14.17	93	6.07	0.96	6.23	−0.01	0.15	−0.29	0.28
Roepke et al. [51]	4 weeks	post T1	IG1(CBT-PPT SB) vs. CG	SWLS	Global	16.6	20	7.78	15.08	36	6.86	0.47	7.20	0.21	0.28	−0.35	0.77
Roepke et al. [51]	4 weeks	post T1	IG2 (SB) vs CG	SWLS	Global	18.56	18	7.16	15.08	36	6.86	0.10	6.96	0.49	0.29	−0.09	1.09
Roepke et al. [51]	6 weeks	post T2	IG1(CBT-PPT SB) vs. CG	SWLS	Global	19.64	11	7.8	14.45	29	6.63	0.07	6.96	0.73	0.36	0.01	1.48
Roepke et al. [51]	6 weeks	post T2	IG2 (SB) vs CG	SWLS	Global	18.42	12	7.6	14.45	29	6.63	0.13	6.92	0.56	0.35	−0.13	1.28
Schefft et al. [48]		pre	IG (unguided) vs. CG	WHO-QoL	PH	59.2	150	15.1	58.9	100	12.8	0.87	14.23	0.02	0.13	−0.23	0.28
Schefft et al. [48]	12 weeks	post T1	IG (unguided) vs. CG	WHO-QoL	PH	61.5	116	16.2	46.3	52	14.1	0.00	15.58	0.97	0.18	0.63	1.32
Schefft et al. [48]	24 weeks	post T2	IG (unguided) vs. CG	WHO-QoL	PH	55.1	62	19.5	57.9	29	17.4	0.49	18.86	−0.15	0.23	−0.60	0.30
Schefft et al. [48]		pre	IG (unguided) vs. CG	WHO-QoL	MH	49.9	150	12.2	49.3	100	14.8	0.74	13.30	0.04	0.13	−0.21	0.30
Schefft et al. [48]	12 weeks	post T1	IG (unguided) vs. CG	WHO-QoL	MH	59.4	116	12.5	36.2	52	12.5	0.00	12.50	1.85	0.20	1.47	2.24
Schefft et al. [48]	24 weeks	post T2	IG (unguided) vs. CG	WHO-QoL	MH	58.4	62	11	37.9	39	14.4	0.00	12.42	1.64	0.24	1.18	2.12
Schefft et al. [48]		pre	IG (unguided) vs. CG	WHO-QoL	SR	61.9	150	18.8	65	100	20.1	0.22	19.33	−0.16	0.13	−0.42	0.09
Schefft et al. [48]	12 weeks	post T1	IG (unguided) vs. CG	WHO-QoL	SR	49.8	116	21	35.3	52	20.9	0.00	20.97	0.69	0.17	0.35	1.03
Schefft et al. [48]	24 weeks	post T2	IG (unguided) vs. CG	WHO-QoL	SR	53.1	62	22.2	45.7	29	21.3	0.13	21.92	0.33	0.23	−0.11	0.79
Schefft et al. [48]		pre	IG (unguided) vs. CG	WHO-QoL	EN	76.3	150	18	78.8	100	12.6	0.20	16.06	−0.16	0.13	−0.41	0.10
Schefft et al. [48]	12 weeks	post T1	IG (unguided) vs. CG	WHO-QoL	EN	67.8	116	14.5	65	52	17.3	0.31	15.41	0.18	0.17	−0.15	0.51
Schefft et al. [48]	24 weeks	post T2	IG (unguided) vs. CG	WHO-QoL	EN	60.6	62	21.4	67	29	15.8	0.11	19.81	−0.32	0.23	−0.77	0.13
Stuart et al. [40]		pre	IG vs. CG	BRFSS	Global	4.9	139	2.11	4.8	150	1.87	0.67	1.99	0.05	0.12	−0.18	0.28
Stuart et al. [40]	8 weeks	post T1	IG vs. CG	BRFSS	Global	6.3	77	1.8	5.7	99	0.76	0.01	1.32	0.45	0.15	0.15	0.76
Stuart et al. [40]	24 weeks	post T2	IG vs. CG	BRFSS	Global	6	82	1.84	5.9	114	1.91	0.71	1.88	0.05	0.14	−0.23	0.34
Wong et al. [54]		pre	IG vs. CG	SF-6D	Global	0.6	39	0.1	0.6	40	0.1	1.00	0.10	0.00	0.23	−0.45	0.45
Wong et al. [54]	9 weeks	post T1	IG vs. CG	SF-6D	Global	0.7	39	0.1	0.7	40	0.1	1.00	0.10	0.00	0.23	−0.45	0.45

Note. Global =overall QoL score. PH = physical health, MH = mental health, SR = social relationships, EN = environment

## Appendix 2

See Table 5.

**Table 5:** Raw and standardized depression data

Study	Time	Measure.	Comparison	Questionnaire	IG			CG			p-Value	Pooled SD	g	S.E.	CI-	CI+
					Mean	n	SD	Mean	n	SD						
Berger et al. [42]		pre	IG1 vs CG	BDI-2	29.80	25	8.60	29.80	26	8.60	1.00	8.60	0.00	0.28	−0.56	0.56
Berger et al. [42]	10 weeks	post T1	IG1 vs CG	BDI-2	20.80	25	13.50	28.50	26	9.40	0.02	11.59	−0.65	0.29	−1.24	−0.09
Berger et al. [42]	24 weeks	post T2	IG1 vs CG	BDI-2	19.40	25	12.90	21.08	26	9.90	0.61	11.47	−0.14	0.28	−0.71	0.42
Jelinek et al. [47]		pre	IG1 vs CG	PHQ-9	10.46	37	4.32	11.00	35	4.27	0.60	4.30	−0.12	0.24	−0.60	0.35
Jelinek et al. [47]		pre	IG2 vs CG	PHQ-9	12.50	32	5.04	11.00	35	4.27	0.20	4.65	0.32	0.25	−0.17	0.81
Jelinek et al. [47]	2 weeks	post T1	IG1 vs CG	PHQ-9	9.73	29	5.46	9.90	32	3.14	0.88	4.40	−0.04	0.26	−0.55	0.47
Jelinek et al. [47]	2 weeks	post T1	IG2 vs CG	PHQ-9	11.37	27	6.15	9.90	32	3.14	0.27	4.76	0.31	0.26	−0.22	0.84
Jelinek et al. [47]	4 weeks	post T2	IG1 vs CG	PHQ-9	8.70	27	5.72	9.26	31	4.14	0.68	4.94	−0.11	0.26	−0.64	0.41
Jelinek et al. [47]	4 weeks	post T2	IG2 vs CG	PHQ-9	11.57	21	5.27	9.26	31	4.14	0.10	4.63	0.49	0.29	−0.08	1.08
Gili et al. [39]		pre	IG (PAPP) vs. CG (iTAU)	PHQ-9	15.79	56	6.21	14.39	57	5.83	0.22	6.02	0.23	0.19	−0.14	0.61
Gili et al. [39]		pre	IG (HLP) vs. CG (iTAU)	PHQ-9	15.80	54	5.31	14.39	57	5.83	0.19	5.58	0.25	0.19	−0.13	0.63
Gili et al. [39]		pre	IG (MP) vs. CG (iTAU)	PHQ-9	15.39	54	5.70	14.39	57	5.83	0.36	5.77	0.17	0.19	−0.20	0.55
Gili et al. [39]	after treatment*	post T1	IG (PAPP) vs. CG (iTAU)	PHQ-9	10.57	56	6.68	12.08	57	6.20	0.22	6.44	−0.23	0.19	−0.61	0.14
Gili et al. [39]	after treatment*	post T1	IG (HLP) vs. CG (iTAU)	PHQ-9	8.98	54	5.90	12.08	57	6.20	0.01	6.06	−0.51	0.19	−0.89	−0.13
Gili et al. [39]	after treatment*	post T1	IG (MP) vs. CG (iTAU)	PHQ-9	9.04	54	6.54	12.08	57	6.20	0.01	6.37	−0.47	0.19	−0.86	−0.10
Gili et al. [39]	24 weeks	post T2	IG (PAPP) vs. CG (iTAU)	PHQ-9	8.62	56	6.11	10.70	57	7.32	0.10	6.75	−0.31	0.19	−0.68	0.07
Gili et al. [39]	24 weeks	post T2	IG (HLP) vs. CG (iTAU)	PHQ-9	9.19	54	6.18	10.70	57	7.32	0.24	6.79	−0.22	0.19	−0.60	0.16
Gili et al. [39]	24 weeks	post T2	IG (MP) vs. CG (iTAU)	PHQ-9	9.02	54	6.65	10.70	57	7.32	0.21	7.00	−0.24	0.19	−0.62	0.14
Gili et al. [39]	48 weeks	post T3	IG (PAPP) vs. CG (iTAU)	PHQ-9	9.75	56	5.64	9.77	57	6.27	0.99	5.97	0.00	0.19	−0.38	0.37
Gili et al. [39]	48 weeks	post T3	IG (HLP) vs. CG (iTAU)	PHQ-9	9.37	54	6.48	9.77	57	6.27	0.74	6.37	−0.06	0.19	−0.44	0.31
Gili et al. [39]	48 weeks	post T3	IG (MP) vs. CG (iTAU)	PHQ-9	9.83	54	6.30	9.77	57	6.27	0.96	6.28	0.01	0.19	−0.37	0.39
Lüdtke et al. [46]		pre	IG vs. CG	PHQ-9	11.61	44	6.14	12.77	44	6.40	0.39	6.27	−0.18	0.21	−0.61	0.24
Lüdtke et al. [46]	4 weeks	post	IG vs. CG	PHQ-9	10.23	35	5.56	10.72	39	6.05	0.72	5.82	−0.08	0.23	−0.55	0.38
Bruhns et al. [53]		pre	IG vs. CG	PHQ-9	11.27	90	5.03	11.10	135	4.32	0.79	4.62	0.04	0.14	−0.23	0.31
Bruhns et al. [53]	4 weeks	post	IG vs. CG	PHQ-9	9.30	90	5.22	10.17	135	4.32	0.19	4.70	−0.18	0.14	−0.45	0.08
Stuart et al. [40]		pre	IG vs. CG	PHQ-9	14.60	148	5.28	14.90	154	5.07	0.61	5.17	−0.06	0.12	−0.28	0.17
Stuart et al. [40]	8 weeks	post T1	IG vs. CG	PHQ-9	8.10	77	4.92	10.90	99	5.08	0.00	5.01	−0.56	0.15	−0.86	−0.25
Stuart et al. [40]	24 weeks	post T2	IG vs. CG	PHQ-9	8.90	82	5.54	9.90	114	5.45	0.21	5.49	−0.18	0.15	−0.47	0.10
Wong et al. [54]		prä	IG vs. CG	BDI-2	13.50	39	4.00	12.30	40	3.60	0.17	3.80	0.31	0.23	−0.14	0.77
Wong et al. [54]	9 weeks	post T1	IG vs. CG	BDI-2	8.80	39	3.80	11.60	40	4.70	0.00	4.28	−0.65	0.23	−1.11	−0.19
Roepke et al. [51]		pre	IG1(CBT-PPT SB) vs. CG	CES-D	34.48	93	9.24	33.07	93	8.81	0.29	9.03	0.16	0.15	−0.13	0.45
Roepke et al. [51]		pre	IG2 (SB) vs CG	CES-D	33.07	97	8.81	33.07	93	8.81	1.00	8.81	0.00	0.15	−0.29	0.29
Roepke et al. [51]	2 weeks	post T1	IG1(CBT-PPT SB) vs. CG	CES-D	25.66	29	12.93	28.34	58	10.60	0.34	11.42	−0.23	0.23	−0.69	0.22
Roepke et al. [51]	2 weeks	post T1	IG2 (SB) vs CG	CES-D	23.77	30	10.81	28.34	58	10.60	0.06	10.67	−0.42	0.23	−0.88	0.02
Roepke et al. [51]	4 weeks	post T2	IG1(CBT-PPT SB) vs. CG	CES-D	23.55	20	13.73	27.36	36	10.63	0.29	11.81	−0.32	0.28	−0.88	0.24
Roepke et al. [51]	4 weeks	post T2	IG2 (SB) vs CG	CES-D	19.06	18	10.30	27.36	36	10.63	0.01	10.52	−0.78	0.30	−1.39	−0.19
Roepke et al. [51]	6 weeks	post T3	IG1(CBT-PPT SB) vs. CG	CES-D	18.73	11	13.19	25.14	29	15.14	0.20	14.65	−0.43	0.36	−1.16	0.29
Roepke et al. [51]	6 weeks	post T3	IG2 (SB) vs CG	CES-D	16.83	12	9.63	25.14	29	15.14	0.04	13.81	−0.59	0.35	−1.31	0.11
Bolier et al. [49]		pre	IG vs. CG	CES-D	16.91	143	4.16	16.67	141	4.12	0.63	4.14	0.06	0.12	−0.18	0.29
Bolier et al. [49]	8 weeks	post T1	IG vs. CG	CES-D	13.67	95	6.69	15.39	119	7.64	0.08	7.23	−0.24	0.14	−0.51	0.03
Bolier et al. [49]	24 weeks	post T2	IG vs. CG	CES-D	13.06	89	7.55	14.94	109	7.48	0.08	7.51	−0.25	0.14	−0.53	0.03
Klein et al. [45]		pre	IG vs. KG	PHQ-9	7.62	192	1.19	7.70	187	1.25	0.52	1.22	−0.07	0.10	−0.27	0.14
Klein et al. [45]	12 weeks	post T1	IG vs KG	PHQ-9	6.29	154	3.54	7.61	155	3.82	0.00	3.68	−0.36	0.11	−0.58	−0.13
Klein et al. [45]	24 weeks	post T2	IG vs KG	PHQ-9	5.84	148	3.84	7.28	141	4.41	0.00	4.13	−0.35	0.12	−0.58	−0.12
Moritz et al. (2011)		pre	IG vs KG	BDI-2	28.81	105	11.11	30.02	105	10.18	0.41	10.66	−0.11	0.14	−0.39	0.16
Moritz et al. (2011)	8 weeks	post T1	IG vs KG	BDI-2	20.51	105	12.22	25.67	105	11.65	0.00	11.94	−0.43	0.14	−0.71	−0.16
Meyer et al. [52]		pre	IG vs. KG	PHQ-9	16.62	78	3.44	17.20	85	3.86	0.31	3.67	−0.16	0.16	−0.47	0.15
Meyer et al. [52]	12 weeks	post T1	IG vs KG	PHQ-9	10.08	61	6.37	13.64	73	6.14	0.00	6.25	−0.57	0.18	−0.92	−0.22
Meyer et al. [52]	24 weeks	post T2	IG vs KG	PHQ-9	11.28	57	6.04	13.39	64	6.59	0.07	6.34	−0.33	0.18	−0.70	0.03

## Appendix 3

The OVID search string was: ((Depress* or depressive disorder) and (internet intervention or internet-based or internetbased psychotherap* or internet-based therap* or internet-based treat* or iCBT or dCBT or digital therap* or digital psychotherap* or digital intervention* or digital treat* or smartphone or smartphone therap* or smartphone psychotherap* or smartphone intervention* or smartphone treat* or mobile therap* or mobile psychotherap* or mobile intervention* or mobile treat* or selfmanagement or self-management intervention or self-management treat*) and (Quality Life or wellbeing)).ti,ab.

The PubMed search string was: ((Depress* [Title]) OR (Depression [MeSH Terms]) OR (depressive disorder [MeSH Terms])) AND ((Internet-based Interventions [Title/Abstract]) OR (internet intervention [Title/Abstract]) OR (internetbased [Title/Abstract]) OR (internet-based psychotherap* [Title/Abstract]) OR (internet-based therap* [Title/Abstract]) OR (internet-based treat* [Title/Abstract]) OR (iCBT [Title/Abstract]) OR (dCBT [Title/Abstract]) OR (digital therap* [Title/Abstract]) OR (digital psychotherap* [Title/Abstract]) OR (digital intervention* [Title/Abstract]) OR (digital treat* [Title/Abstract]) OR (smartphone [Title/Abstract]) OR (smartphone therap* [Title/Abstract]) OR (smartphone psychotherap* [Title/Abstract]) OR (smartphone intervention* [Title/Abstract]) OR (smartphone treat* [Title/Abstract]) OR (mobile therap* [Title/Abstract]) OR (mobile psychotherap* [Title/Abstract]) OR (mobile intervention* [Title/Abstract]) OR (mobile treat* [Title/Abstract]) OR (self-management [Title/Abstract]) OR (self-management intervention [Title/Abstract]) OR (self-management treat* [Title/Abstract])) AND ((QoL [Title/Abstract]) OR (quality of life [Title/Abstract]) OR (wellbeing [Title/Abstract])) OR (wellbeing [Title/Abstract]).

## References

[1] Institute of Health Metrics and Evaluation. (2021). Global health data exchange (GHDx).

[2] Richter, D., Wall, A., Bruen, A., & Whittington, R. (2019). Is the global prevalence rate of adult mental illness increasing? Systematic review and meta-analysis. *Acta Psychiatrica Scandinavica,**140*(5), 393–407. 10.1111/acps.13083 31393996 10.1111/acps.13083

[3] Papakostas, G. I., Petersen, T., Mahal, Y., Mischoulon, D., Nierenberg, A. A., & Fava, M. (2004). Quality of life assessments in major depressive disorder: A review of the literature. *General Hospital Psychiatry,**26*(1), 13–17. 10.1016/j.genhosppsych.2003.07.004 14757297 10.1016/j.genhosppsych.2003.07.004

[4] Rapaport, M. H., Clary, C., Fayyad, R., & Endicott, J. (2005). Quality-of-life impairment in depressive and anxiety disorders. *American Journal of Psychiatry,**162*(6), 1171–1178. 10.1176/appi.ajp.162.6.1171 15930066 10.1176/appi.ajp.162.6.1171

[5] World Health Organization. (1995). The World Health Organization quality of life assessment (WHO/QOL): Position paper from the World Health Organization. *Social Science & Medicine,**41*(10), 1403–1409. 10.1016/0277-9536(95)00112-K 8560308 10.1016/0277-9536(95)00112-k

[6] Ishak, W. W., Wen, R. Y., Naghdechi, L., Vanle, B., Dang, J., Knosp, M., Dascal, J., Marcia, L., Gohar, Y., Eskander, L., et al. (2018). Pain and depression: A systematic review. *Harvard Review of Psychiatry,**26*(6), 352–363. 10.1097/HRP.0000000000000198 30407234 10.1097/HRP.0000000000000198

[7] LeMoult, J., & Gotlib, I. H. (2019). Depression: A cognitive perspective. *Clinical Psychology Review,**69*, 51–66. 10.1016/j.cpr.2018.06.008 29961601 10.1016/j.cpr.2018.06.008 PMC11884012

[8] Mu, W., Luo, J., Rieger, S., Trautwein, U., & Roberts, B. W. (2019). The relationship between self-esteem and depression when controlling for neuroticism. *Collabra Psychology*. 10.1525/collabra.204

[9] Brechan, I., & Kvalem, I. L. (2015). Relationship between body dissatisfaction and disordered eating: Mediating role of self-esteem and depression. *Eating Behaviors,**17*, 49–58. 10.1016/j.eatbeh.2014.12.008 25574864 10.1016/j.eatbeh.2014.12.008

[10] Guan, N., Guariglia, A., Moore, P., Xu, F., & Al-Janabi, H. (2022). Financial stress and depression in adults: A systematic review. *PLoS ONE,**17*(2), e0264041. 10.1371/journal.pone.0264041 35192652 10.1371/journal.pone.0264041 PMC8863240

[11] Evans, S., Banerjee, S., Leese, M., & Huxley, P. (2007). The impact of mental illness on quality of life: A comparison of severe mental illness, common mental disorder and healthy population samples. *Quality of Life Research,**16*, 17–29. 10.1007/s11136-006-9002-6 17036252 10.1007/s11136-006-9002-6

[12] Ishak, W. W., Greenberg, J. M., & Cohen, R. M. (2013). Predicting relapse in major depressive disorder using patient-reported outcomes of depressive symptom severity, functioning, and quality of life in the individual burden of illness index for depression (IBI-D). *Journal of Affective Disorders,**151*(1), 59–65. 10.1016/j.jad.2013.05.056 23790554 10.1016/j.jad.2013.05.048 PMC4167729

[13] Ishak, W. W., Greenberg, J. M., Balayan, K., Kapitanski, N., Jeffrey, J., Fathy, H., Fakhry, H., & Rapaport, M. H. (2011). Quality of life: The ultimate outcome measure of interventions in major depressive disorder. *Harvard Review of Psychiatry,**19*(5), 229–239. 10.3109/10673229.2011.614099 21916825 10.3109/10673229.2011.614099

[14] Fava, G. A., Ruini, C., & Belaise, C. (2007). The concept of recovery in major depression. *Psychological Medicine,**37*(3), 307–317. 10.1017/S0033291706008981 17311684 10.1017/S0033291706008981

[15] González-Blanch, C., Hernández-de-Hita, F., Muñoz-Navarro, R., Ruíz-Rodríguez, P., Medrano, L. A., & Cano-Vindel, A. (2018). The association between different domains of quality of life and symptoms in primary care patients with emotional disorders. *Scientific Reports,**8*(1), 11180. 10.1038/s41598-018-28995-6 30046118 10.1038/s41598-018-28995-6 PMC6060102

[16] Shumye, S., Belayneh, Z., & Mengistu, N. (2019). Health related quality of life and its correlates among people with depression attending outpatient department in Ethiopia: A cross sectional study. *Health and Quality of Life Outcomes,**17*, 169. 10.1186/s12955-019-1233-7 31703701 10.1186/s12955-019-1233-7 PMC6839081

[17] Cuijpers, P., Karyotaki, E., Weitz, E., Andersson, G., Hollon, S. D., & van Straten, A. (2014). The effects of psychotherapies for major depression in adults on remission, recovery and improvement: A meta-analysis. *Journal of Affective Disorders,**159*, 118–126. 10.1016/j.jad.2014.02.026 24679399 10.1016/j.jad.2014.02.026

[18] IsHak, W. W., Mirocha, J., James, D., Tobia, G., Vilhauer, J., Fakhry, H., Pi, S., Hanson, E., Nashawati, R., Peselow, E. D., et al. (2015). Quality of life in major depressive disorder before/after multiple steps of treatment and one-year follow-up. *Acta Psychiatrica Scandinavica,**131*(1), 51–60. 10.1111/acps.12291 24954156 10.1111/acps.12301 PMC4267902

[19] Hofmann, S. G., Curtiss, J., Carpenter, J. K., & Kind, S. (2017). Effect of treatments for depression on quality of life: A meta-analysis. *Cognitive Behaviour Therapy,**46*(4), 265–286. 10.1080/16506073.2017.1304445 28440699 10.1080/16506073.2017.1304445 PMC5663193

[20] Kolovos, S., Kleiboer, A., & Cuijpers, P. (2016). Effect of psychotherapy for depression on quality of life: Meta-analysis. *The British Journal of Psychiatry,**209*(6), 460–468. 10.1192/bjp.bp.115.175059 27539296 10.1192/bjp.bp.115.175059

[21] Saxena, S., Thornicroft, G., Knapp, M., & Whiteford, H. (2007). Resources for mental health: Scarcity, inequity, and inefficiency. *The Lancet,**370*(9590), 878–889. 10.1016/S0140-6736(07)61239-2 10.1016/S0140-6736(07)61239-2 17804062

[22] Linardon, J., Cuijpers, P., Carlbring, P., Messer, M., & Fuller-Tyszkiewicz, M. (2019). The efficacy of app-supported smartphone interventions for mental health problems: A meta-analysis of randomized controlled trials. *World Psychiatry,**18*(3), 325–336. 10.1002/wps.20673 31496095 10.1002/wps.20673 PMC6732686

[23] Karyotaki, E., Riper, H., Twisk, J., Hoogendoorn, A., Kleiboer, A., Mira, A., Mackinnon, A., Meyer, B., Botella, C., Littlewood, E., et al. (2017). Efficacy of self-guided internet-based cognitive behavioral therapy in the treatment of depressive symptoms: A meta-analysis of individual participant data. *JAMA Psychiatry,**74*(4), 351–359. 10.1001/jamapsychiatry.2017.0044 28241179 10.1001/jamapsychiatry.2017.0044

[24] Weisel, K. K., Fuhrmann, L. M., Berking, M., Baumeister, H., Cuijpers, P., & Ebert, D. D. (2019). Standalone smartphone apps for mental health: A systematic review and meta-analysis. *Npj Digital Medicine,**2*(1), 118. 10.1038/s41746-019-0188-8 31815193 10.1038/s41746-019-0188-8 PMC6889400

[25] Moshe, I., Terhorst, Y., Philippi, P., Domhardt, M., Cuijpers, P., Cristea, I., Pulkki-Råback, L., Baumeister, H., & Sander, L. B. (2021). Digital interventions for the treatment of depression: A meta-analytic review. *Psychological Bulletin,**147*(8), 749–786. 10.1037/bul0000334 34898233 10.1037/bul0000334

[26] Firth, J., Torous, J., Nicholas, J., Carney, R., Pratap, A., Rosenbaum, S., & Sarris, J. (2017). The efficacy of smartphone-based mental health interventions for depressive symptoms: A meta-analysis of randomized controlled trials. *World Psychiatry,**16*(3), 287–298. 10.1002/wps.20472 28941113 10.1002/wps.20472 PMC5608852

[27] Wu, A., Scult, M. A., Barnes, E. D., Betancourt, J. A., Falk, A., & Gunning, F. M. (2021). Smartphone apps for depression and anxiety: A systematic review and meta-analysis of techniques to increase engagement. *NPJ Digital Medicine,**4*(1), 20. 10.1038/s41746-021-00463-8 33574573 10.1038/s41746-021-00386-8 PMC7878769

[28] Yang, D., Hur, J.-W., Kwak, Y. B., & Choi, S.-W. (2018). A systematic review and meta-analysis of applicability of web-based interventions for individuals with depression and quality of life impairment. *Psychiatry Investigation,**15*(8), 759–767. 10.30773/pi.2018.06.30 30048585 10.30773/pi.2018.03.15 PMC6111215

[29] Hrynyschyn, R., & Dockweiler, C. (2021). Effectiveness of smartphone-based cognitive behavioral therapy among patients with major depression: Systematic review of health implications. *JMIR mHealth and uHealth,**9*(2), e24703. 10.2196/24703 33565989 10.2196/24703 PMC7904402

[30] Fadipe, M. F., Aggarwal, S., Johnson, C., & Beauchamp, J. E. S. (2023). Effectiveness of online cognitive behavioural therapy on quality of life in adults with depression: A systematic review. *Journal of Psychiatric and Mental Health Nursing,**30*(5), 885–898. 10.1111/jpm.12920 37010913 10.1111/jpm.12924

[31] Maj, A., Michalak, N., Graczykowska, A., & Andersson, G. (2023). The effect of internet-delivered cognitive behavioral therapy for depression and anxiety on quality of life: A meta-analysis of randomized controlled trials. *Internet Interventions,**33*, 100654. 10.1016/j.invent.2023.100654 37555075 10.1016/j.invent.2023.100654 PMC10404731

[32] Moher, D., Liberati, A., Tetzlaff, J., Altman, D. G., & PRISMA Group. (2009). Preferred reporting items for systematic reviews and meta-analyses: The PRISMA statement. *Annals of Internal Medicine,**151*(4), 264–269. 10.7326/0003-4819-151-4-200908180-00135 19622511 10.7326/0003-4819-151-4-200908180-00135

[33] Sterne, J. A. C., Savović, J., Page, M. J., Elbers, R. G., Blencowe, N. S., Boutron, I., Cates, C. J., Cheng, H.-Y., Corbett, M. S., Eldridge, S. M., et al. (2019). RoB 2: A revised tool for assessing risk of bias in randomised trials. *BMJ,**366*, l4898. 10.1136/bmj.l4898 31462531 10.1136/bmj.l4898

[34] Torous, J., Lipschitz, J., Ng, M., & Firth, J. (2020). Dropout rates in clinical trials of smartphone apps for depressive symptoms: A systematic review and meta-analysis. *Journal of Affective Disorders,**263*, 413–419. 10.1016/j.jad.2019.11.167 31969272 10.1016/j.jad.2019.11.167

[35] Schünemann, H. J., Best, D., Vist, G., Oxman, A. D., GRADE Working Group. (2003). Letters, numbers, symbols and words: How to communicate grades of evidence and recommendations. *Canadian Medical Association Journal,**169*(7), 677–680. 14517128 PMC202287

[36] Hong, C., Salanti, G., Morton, S. C., Riley, R. D., Chu, H., Kimmel, S. E., & Chen, Y. (2020). Testing small study effects in multivariate meta-analysis. *Biometrics,**76*(4), 1240–1250. 10.1111/biom.13220 32720712 10.1111/biom.13342 PMC7736122

[37] Forster, M. R. (2000). Key concepts in model selection: Performance and generalizability. *Journal of Mathematical Psychology,**44*(1), 205–231. 10.1006/jmps.1999.1284 10733865 10.1006/jmps.1999.1284

[38] Borenstein, M., Hedges, L. V., Higgins, J. P., & Rothstein, H. R. (2021). *Introduction to meta-analysis*. Wiley.

[39] Gili, M., Castro, A., García-Palacios, A., Garcia-Campayo, J., Mayoral-Cleries, F., Botella, C., Roca, M., Barceló-Soler, A., Hurtado, M. M., Navarro, M. T., et al. (2020). Efficacy of three low-intensity, internet-based psychological interventions for the treatment of depression in primary care: Randomized controlled trial. *Journal of Medical Internet Research,**22*(6), e15845. 10.2196/15845 32501276 10.2196/15845 PMC7305559

[40] Stuart, R., Fischer, H., Leitzke, A. S., Becker, D., Saheba, N., & Coleman, K. J. (2022). The effectiveness of internet-based cognitive behavioral therapy for the treatment of depression in a large real-world primary care practice: A randomized trial. *Permanente Journal,**26*(3), 1–10. 10.7812/TPP/21.276 10.7812/TPP/21.183 PMC9683746 35939620

[41] Cumpston, M., Li, T., Page, M. J., Chandler, J., Welch, V. A., Higgins, J. P. T., & Thomas, J. (2019). Updated guidance for trusted systematic reviews: A new edition of the cochrane handbook for systematic reviews of interventions. *The Cochrane Database of Systematic Reviews*, 10, ED000142. 10.1002/14651858.ED000142 10.1002/14651858.ED000142 PMC10284251 31643080

[42] Berger, T., Hämmerli, K., Gubser, N., Andersson, G., & Caspar, F. (2011). Internet-based treatment of depression: A randomized controlled trial comparing guided with unguided self-help. *Cognitive Behaviour Therapy,**40*(4), 251–266. 10.1080/16506073.2011.616531 22060248 10.1080/16506073.2011.616531

[43] Moritz, S., Schilling, L., Hauschildt, M., Schröder, J., & Treszl, A. (2012). A randomized controlled trial of internet-based therapy in depression. *Behaviour Research and Therapy,**50*(7–8), 513–521. 10.1016/j.brat.2012.04.006 22677231 10.1016/j.brat.2012.04.006

[44] Farrer, L., Christensen, H., Griffiths, K. M., Mackinnon, A., & Batterham, P. J. (2012). Web-based cognitive behavior therapy for depression with and without telephone tracking in a national helpline: Secondary outcomes from a randomized controlled trial. *Journal of Medical Internet Research,**14*(3), e1859. 10.2196/jmir.1859 10.2196/jmir.1859 PMC3414870 22738715

[45] Klein, J. P., Berger, T., Schröder, J., Späth, C., Meyer, B., Caspar, F., Lutz, W., Arndt, A., Greiner, W., Gräfe, V., et al. (2016). Effects of a psychological internet intervention in the treatment of mild to moderate depressive symptoms: Results of the EVIDENT study, a randomized controlled trial. *Psychotherapy and Psychosomatics,**85*(4), 218–228. 10.1159/000445355 27230863 10.1159/000445355 PMC8117387

[46] Lüdtke, T., Pult, L. K., Schröder, J., Moritz, S., & Bücker, L. (2018). A randomized controlled trial on a smartphone self-help application (Be Good to Yourself) to reduce depressive symptoms. *Psychiatry Research,**269*, 753–762. 10.1016/j.psychres.2018.08.113 30273901 10.1016/j.psychres.2018.08.113

[47] Jelinek, L., Arlt, S., Moritz, S., Schröder, J., Westermann, S., & Cludius, B. (2020). Brief web-based intervention for depression: Randomized controlled trial on behavioral activation. *Journal of Medical Internet Research,**22*(3), e15312. 10.2196/15312 32213470 10.2196/15312 PMC7146239

[48] Schefft, C., Krämer, R., Haaf, R., Jedeck, D., Schumacher, A., & Köhler, S. (2024). Evaluation of the internet-based intervention “Selfapy” in participants with unipolar depression and the impact on quality of life: A randomized, parallel group study. *Quality of Life Research,**33*(5), 1275–1286. 10.1007/s11136-024-03606-2 38403818 10.1007/s11136-024-03606-2 PMC11045620

[49] Bolier, L., Haverman, M., Kramer, J., Westerhof, G. J., Riper, H., Walburg, J. A., Boon, B., & Bohlmeijer, E. (2013). An internet-based intervention to promote mental fitness for mildly depressed adults: Randomized controlled trial. *Journal of Medical Internet Research,**15*(9), e2603. 10.2196/jmir.2603 10.2196/jmir.2603 PMC3929047 24041479

[50] Crisp, D., Griffiths, K., Mackinnon, A., Bennett, K., & Christensen, H. (2014). An online intervention for reducing depressive symptoms: Secondary benefits for self-esteem, empowerment and quality of life. *Psychiatry Research,**216*(1), 60–66. 10.1016/j.psychres.2014.01.041 24534125 10.1016/j.psychres.2014.01.041

[51] Roepke, A. M., Jaffee, S. R., Riffle, O. M., McGonigal, J., Broome, R., & Maxwell, B. (2015). Randomized controlled trial of SuperBetter, a smartphone-based/internet-based self-help tool to reduce depressive symptoms. *Games for Health Journal,**4*(3), 235–246. 10.1089/g4h.2014.0046 26182069 10.1089/g4h.2014.0046

[52] Meyer, B., Bierbrodt, J., Schröder, J., Berger, T., Beevers, C. G., Weiss, M., Jacob, G., Späth, C., Andersson, G., Lutz, W., et al. (2015). Effects of an internet intervention (Deprexis) on severe depression symptoms: Randomized controlled trial. *Internet Interventions,**2*(1), 48–59. 10.1016/j.invent.2014.12.003

[53] Bruhns, A., Lüdtke, T., Moritz, S., & Bücker, L. (2021). A mobile-based intervention to increase self-esteem in students with depressive symptoms: Randomized controlled trial. *JMIR mHealth and uHealth,**9*(7), e26498. 10.2196/26498 34255711 10.2196/26498 PMC8314153

[54] Wong, V.W.-H., Ho, F.Y.-Y., Shi, N.-K., Tong, J.T.-Y., Chung, K.-F., Yeung, W.-F., Ng, C. H., Oliver, G., & Sarris, J. (2021). Smartphone-delivered multicomponent lifestyle medicine intervention for depressive symptoms: A randomized controlled trial. *Journal of Consulting and Clinical Psychology,**89*(12), 970–979. 10.1037/ccp0000705 35025538 10.1037/ccp0000695

[55] De Boer, M. R., Waterlander, W. E., Kuijper Gao, K., Su, M., Sweet, J., & Calabrese, J. R. (2019). Correlation between depression/anxiety symptom severity and quality of life in patients with major depressive disorder or bipolar disorder. *Journal of affective disorders,**244*, 9–15. 30292023 10.1016/j.jad.2018.09.063

[56] Pietrzykowski, T., Watt, S., Hills, S., Short, K., Wicks, A., & Sharp, J. (2024). Establishing minimally important differences in WHOQOL-BREF scores in adults with neurofibromatosis. *Quality of Life Research,**33*(2), 335–345. 10.1007/s11136-023-03434-937906345

[57] Pickard, A. S., Neary, M. P., & Cella, D. (2007). Estimation of minimally important differences in EQ-5D utility and VAS scores in cancer. *Health and Quality of Life Outcomes,**5*, 70. 10.1186/1477-7525-5-70 18154669 10.1186/1477-7525-5-70 PMC2248572

[58] Ware, J. E., Kosinski, M., & Keller, S. D. (1995). *SF-12: How to score the SF-12 physical and mental health summary scales* (2nd ed.). QualityMetric Inc.

[59] Pavot, W., & Diener, E. (2008). The satisfaction with life scale and the emerging construct of life satisfaction. *The Journal of Positive Psychology,**3*(2), 137–152. 10.1080/17439760701756946

[60] Cuijpers, P., Noma, H., Karyotaki, E., Cipriani, A., & Furukawa, T. A. (2019). Effectiveness and acceptability of cognitive behavior therapy delivery formats in adults with depression: A network meta-analysis. *JAMA Psychiatry,**76*(7), 700–707. 10.1001/jamapsychiatry.2019.0268 30994877 10.1001/jamapsychiatry.2019.0268 PMC6583673

[61] Bur, O. T., Krieger, T., Moritz, S., Klein, J. P., & Berger, T. (2022). Optimizing the context of support of web-based self-help in individuals with mild to moderate depressive symptoms: A randomized full factorial trial. *Behaviour Research and Therapy,**152*, 104070. 35306266 10.1016/j.brat.2022.104070

[62] Mouratidis, K. (2021). How COVID-19 reshaped quality of life in cities: A synthesis and implications for urban planning. *Land Use Policy,**111*, 105772. 10.1016/j.landusepol.2021.105772 34566233 10.1016/j.landusepol.2021.105772 PMC8456312

